# Sublytic C5b-9 Induces CCL3/4 Production and Macrophage Accumulation in Thy-1N Rats via PKC-α/p65/IRF-8 Axis

**DOI:** 10.7150/ijbs.69652

**Published:** 2022-05-01

**Authors:** Wenbo Wang, Baomei Qian, Chenhui Zhao, Mingyu Peng, Longfei Liu, Mengxiao Xie, Na Peng, Qingling He, Shuai Ying, Yufeng Zhu, Tao Wang, Dajun Hu, Dan Zhao, Jing Zhang, Yingwei Wang, Wen Qiu

**Affiliations:** 1 Department of Immunology, and Key Laboratory of Immunological Environment and Disease, Nanjing Medical University, Nanjing, Jiangsu 211166, China; 2 Department of Medicine, First Affiliated Hospital of Nanjing Medical University, Nanjing, Jiangsu 210029, China; 3 Department of Rheumatology and Nephrology, The Second People's Hospital of China Three Gorges University, Yichang, China; 4 Clinical Medical Science of the First Clinical Medical College, Nanjing Medical University, Nanjing, Jiangsu 211166, China; 5 Department of Nephrology, The Affiliated Jiangning Hospital of Nanjing Medical University, Nanjing, Jiangsu 211100, China; 6 Key Laboratory of Antibody Technology of Ministry of Health, Nanjing Medical University, Nanjing, Jiangsu 211166, China

**Keywords:** mesangioproliferative glomerulonephritis, Thy-1 nephritis, sublytic C5b-9, IRF-8, CCL3/4, macrophage

## Abstract

Mesangioproliferative glomerulonephritis (MsPGN) is a common human kidney disease. Rat Thy-1 nephritis (Thy-1N) is an animal model widely used for the study of MsPGN. Thy-1N is not only sublytic C5b-9-dependent, but also related to pro-inflammatory cytokine production and macrophage (Mφ) accumulation in rat renal tissues. In this study, we found that the expression or phosphorylation of chemokine CCL3/4, CD68 (Mφ marker), IRF-8, PKC-α and NF-κB-p65 (p65) were all up-regulated both in the renal tissues of Thy-1N rats (*in vivo*) and in the glomerular mesangial cells (GMCs) upon sublytic C5b-9 stimulation (*in vitro*). Further experiments* in vitro* revealed that the phosphorylated PKC-α (p-PKC-α) could promote p65 phosphorylation, and then p-p65 enhanced IRF-8 expression through binding to IRF-8 promotor (-591 ~ -582 nt and -299 ~ -290 nt). Additionally, up-regulation or silencing of IRF-8 gene promoted or reduced CCL3/4 production, and then regulated Mφ chemotaxis. The underlying mechanism involved in IRF-8 binding to CCL3 promoter (-249 ~ -236 nt), which resulted in CCL3 gene transcription. The experiments *in vivo* showed that knockdown of renal PKC-α, p65, IRF-8 and CCL3/4 genes could inhibit CCL3/4 production, Mφ accumulation, GMC proliferation and proteinuria of Thy-1N rats. Furthermore, p-PKC-α, p-p65, IRF-8, CCL3/4 expression and Mφ accumulation were also increased in the renal tissues of MsPGN patients. Collectively, these findings indicate that sublytic C5b-9 induces CCL3/4 production and Mφ accumulation via PKC-α/p65/IRF-8 axis, and finally aggravates the pathological changes of MsPGN.

## Introduction

Mesangioproliferative glomerulonephritis (MsPGN) is a chronic kidney disease in human 1-3. The mechanism of MsPGN involves in complement activation, inflammatory cell (e.g. macrophage, Mφ) infiltration, pro-inflammatory cytokine generation, ultimately leading to glomerular mesangial cell (GMC) proliferation, extracellular matrix (ECM) secretion and renal tissue fibrosis 1-5. Although many studies have proved that the deposition of complement C5b-9 and the production of pro-inflammatory cytokines including chemokines are increased simultaneously in the glomeruli of MsPGN patients 1, 6, 7, the relationship between C5b-9 formation and cytokine expression in MsPGN has not been fully illuminated.

Rat Thy-1 nephritis (Thy-1N) is an animal model for studying MsPGN 8-10. Similar to human MsPGN, Thy-1N undergoes complement activation and a series of pathological changes including renal inflammation, GMC proliferation and ECM accumulation. Thy-1N is C5b-9-dependent especially sublytic C5b-9-dependent and neutrophil-independent 11, 12. Our previous studies have disclosed that rat GMCs stimulated with sublytic C5b-9 can increase the production of not only pro-inflammatory cytokines (IL-6, IL-23, IL-36α) but also chemokines (MCP-1/CCL2 and RANTES), and the expression of these cytokines is correlated with the up-regulation of several transcriptional factors (i.e. CEBPβ and KLF6) in the GMCs 13-15.

Reportedly, interferon regulatory factor-8 (IRF-8), as a transcriptional factor, can promote IL-12, IL-23, CCL6 and CCL9 expression and induce inflammatory diseases such as experimental autoimmune encephalomyelitis (EAE) 16-18. In the early stage of this study, we found that the expression levels of IRF-8 and chemokine C-C motif ligand 3/4 (CCL3/4) were obviously increased both in the renal tissues of Thy-1N rats and in the GMCs stimulated with sublytic C5b-9, and further bioinformatics prediction indicates that CCL3 gene promotor contains some IRF-8 binding sites. Therefore, the possible effects of IRF-8 on CCL3/4 production in Thy-1N need to be determined.

As we know, protein kinase C-alpha (PKC-α) and nuclear factor kappa-B-p65 (NF-κB-p65, p65) play important regulatory roles in some inflammatory pathological changes 19-24. PKC-α activation (phosphorylated PKC-α, p-PKC-α) can phosphorylate its downstream substrates and cause the activation of various signaling molecules e.g. Akt and mitogen-activated protein kinase (MAPK) 20-23, 25, and the phosphorylated p65 (p-p65) can also induce itself nuclear translocation and promote IL-6 and iNOS expression 19, 24, 25. In the present study, we found that the phosphorylation of PKC-α and p65 was significantly elevated, and the phase of PKC-α/p65 phosphorylation was slightly earlier than that of IRF-8 and CCL3/4 expression, however whether PKC-α/p65 phosphorylation can regulate IRF-8 and CCL3/4 expression and Mφ accumulation in Thy-1N remains unclear.

In this study, we examined CCL3/4 production, Mφ chemotaxis and IRF-8 expression, and then investigated the roles of IRF-8 in CCL3/4 production in the GMCs treated with sublytic C5b-9, and the effects of CCL3/4 on Mφ chemotaxis. Meantime, the levels of p-PKC-α and p-p65 and their regulatory roles in IRF-8 expression were assessed. Furthermore, the effects of silencing renal PKC-α, p65, IRF-8 and CCL3/4 genes on the corresponding protein expression or phosphorylation, Mφ accumulation, GMC lesion and proteinuria of Thy-1N rats were evaluated. Besides, the expression of p-PKC-α, p-p65, IRF-8, CCL3/4 and the accumulation of Mφ in the renal tissues of MsPGN patients were also observed, and the correlation of these parameters was analyzed.

## Materials and Methods

### Reagents

Abs against t-ERK1/2 (4695), p-ERK1/2 (Thr202/Tyr204, 4370), t-p38 MAPK (9212), p-p38 MAPK (Thr180/Tyr182, 4511), t-JNK (9258), p-JNK (Thr183/Tyr185, 4668), t-p65 (8242), p-p65 (Ser536, 3033), t-STAT3 (9139), p-STAT3 (Tyr705, 9145), HRP-conjugated anti-rabbit IgG (Light-Chain Specific, 93702) were purchased from Cell Signaling Technology (USA). Abs against t-PKC-α (ab32376), p-PKC-α (Thr638, ab32502), CD68 (ab31630 and ab955), CCL3 (A7568), CCL4 (A1671), CCR1 (A18341) and CCR5 (A20261) were supplied by Abcam (UK) and Abclone (China), respectively. Ab against IRF-8 (sc-365042) and p-p65 (Ser536, sc-136548) were from Santa Cruz Biotechnology (USA). Ab against β-actin (BS6007M), HRP-conjugated anti-rabbit IgG (BS13278) and anti-mouse IgG (BS12478) were supplied by Bioworld Technology (China). CCL3 neutralizing antibody (MAB66252) and CCL4 neutralizing antibody (PA1-29036) were from R&D Systems (USA) and Thermo Fisher Scientific (USA), respectively. A biotin conjugation kit (ab201795) and Streptavidin-HRP (ab7403) was supplied by Abcam. An EZ-ChIP^TM^ chromatin immunoprecipitation kit was from Millipore (USA). The inhibitors against ERK1/2 (U0126), p38 MAPK (SB203580), JNK (SP600125), PKC-α (Bisindolylmaleimide I, BIS), NF-κB-p65 (BAY 11-7082), STAT3 (Stattic) and cell counting kit-8 (CCK-8) were obtained from MedChemExpress (USA).

Rabbit polyclonal antibodies against the Thy-1 antigen (Thy-1 antibody, Thy-1 Ab) were prepared as previously published 26, 27. Normal human sera (NHS) from 30 healthy adult donors were pooled as a source of complement, and part of pooled NHS was incubated at 56ºC for 30 min to obtain heat-inactivated human serum (HIS). Human C6-deficient serum (C6DS) and complement C6 were obtained from Sigma-Aldrich (USA) and Sino Biological Inc. (China), respectively.

### MsPGN patient renal sample collection

The renal specimens from the patients who underwent kidney biopsy or resection operation were collected at First Affiliated Hospital of Nanjing Medical University. The patients with the MsPGN diagnosis were enrolled (n=20), the adjacent tissues from the unaffected pole of kidneys of patients with renal cell carcinomas were collected as controls (n=22). All specimens contained at least 10 glomeruli available for scoring. All patients signed an informed consent document. The procedures were approved by the Ethics Committee of Nanjing Medical University.

### Animals and cells

Male Sprague-Dawley (SD) rats were purchased from the Animal Core Facility of Nanjing Medical University (Nanjing, China). Animal experiments were approved by the Institutional Animal Care and Use Committee of Nanjing Medical University and performed in accordance with the guidelines for the care and use of laboratory animals. Rat GMC cell line (HBZY-1) and alveolar Mφ cell line (NR8383) were provided by China Centre for Type Culture Collection (China) and the Cell Bank of Chinese Academy of Sciences (China) respectively.

### Thy-1N induction

Male SD rats (180-200g) were given Thy-1 Ab (0.75ml/100g) by a single i.v. injection to induce Thy-1N. Control rats were injected with normal rabbit sera (NRS, 0.75ml/100g, i.v.). The rat renal cortex was obtained by sacrifice at different time points, and saved for further detection of corresponding mRNA or protein expression.

### Cell culture and sublytic C5b-9 stimulation

Rat GMCs were cultured in MEM plus 10% fetal bovine serum (FBS) as previously described 28, 29. In this study, 5% Thy-1 Ab and 4% NHS were used to form sublytic C5b-9 as reported previously 11, 28. In addition, cells were treated as follows: (1) MEM, (2) 5% Thy-1 Ab, (3) 5% Thy-1 Ab + 4% HIS, (4) 5% Thy-1 Ab + 4% C6DS, (5) 5% Thy-1 Ab + 4% C6DS + 2mg/L C6, (6) 5% Thy-1 Ab + 4% NHS. Besides, rat alveolar Mφ cell line was maintained in Dulbecco modified Eagle's medium (DMEM) supplemented with 10% FBS.

### Transwell chemotaxis experiment

An upper chamber (Corning, USA) containing rat alveolar Mφ cell line was separated by a membrane from a lower chamber containing GMCs following different treatment. Chemotaxis of Mφ from the upper chamber into the lower chamber was induced by GMC-released chemokines in the lower chamber. The crystal violet staining of the membrane was done to visualize the Mφ undergoing chemotaxis.

### Expression plasmid construction

The expression plasmids of wide type (WT) PKC-α, p65 and IRF-8 namely pIRES2-PKC-α-WT, pIRES2-p65-WT, pIRES2-IRF-8-WT were constructed by inserting the coding sequences of rat PKC-α, p65, and IRF-8 genes into pIRES2-EGFP vector (Clontech, USA), respectively. Briefly, PKC-α, p65 and IRF-8 genes were amplified by polymerase chain reaction (PCR) from GMC cDNA. The amplified products and plasmids were digested with restriction enzymes, and then ligated with T4 DNA ligase. The plasmids of active and inactive mutant PKC (pIRES2-PKC-α-A25E and pIRES2-PKC-α-K368R) as well as the plasmids of active and inactive mutant p65 (pIRES2-p65-S535D and pIRES2-p65-S535A) were constructed by General Biosystems (China).

### shRNA plasmid construction

To silence rat PKC-α, p65 and IRF-8 genes, the plasmids of shPKC-α, shp65 and shIRF-8 were constructed with pGPU6-GFP (GenePharma, China). Meanwhile, the control scrambled shRNA plasmid (shCTR) was constructed. The most effective shRNAs were selected and the sequences are as follows: shPKC-α, 5'-CCAAGAGGAGGGTGAATACTA-3'; shp65, 5'-GGAGTACCCTGAAGCTATAAC-3'; shIRF-8, 5'-GCTGGACATTTCCGAGCCATA-3'; shCTR, 5'-TTCTCCGAACGTGTCACGT-3'.

### Cellular transfection

Plasmids were transfected into cultured GMCs with Invitrogen Neon^TM^ transfection system according to the procedures 30. The transfection efficiency of plasmids was observed by GFP expression in cells (Figure S1).

### Reverse transcription-PCR (RT-PCR) and quantitative real-time PCR (qPCR)

Total RNA was isolated from rat renal cortex or GMCs with TRIzol, and the RNA was reverse transcribed into cDNA by Hifair® Ⅱ 1st Strand cDNA Synthesis Kit (Yeasen, China), HiScript Reverse Transcriptase or HiScript II Q RT SuperMix (Vazyme, China)**.** Regular PCR was done with 2 × Taq Master Mix (Vazyme). The data were normalized to GAPDH. The qPCR amplification was performed with AceQ qPCR SYBR Green Master Mix (Vazyme) or Hieff® qPCR SYBR Green Master Mix (Yeasen) in an ABI StepOnePlus. The data were normalized to β-actin, and relative gene expression was calculated using the 2^-ΔΔCT^ method. Relative primer sequences are provided in Table S1.

### Immunoblotting (IB) analysis

Cells and tissues were lysed with RIPA lysis buffer. Equal qualities of protein were separated in SDS-PAGE gel. Protein IB experiments were performed as previously described 11. Primary Abs against t-ERK1/2 (4695), p-ERK1/2 (4370), t-p38 MAPK (9212), p-p38 MAPK (4511), t-JNK (9258), p-JNK (4668), t-p65 (8242), p-p65 (3033), t-STAT3 (9139), p-STAT3 (9145), t-PKC-α (ab32376), p-PKC-α (ab32502), IRF-8 (sc-365042), CCR1 (A18341), CCR5 (A20261), β-actin (BS6007M), and HRP-conjugated anti-mouse or anti-rabbit IgG were used to detect the protein expression or phosphorylation. The bands were shown through ECL detection system and captured by GE Amersham Imager 600.

### Enzyme-linked immunosorbent assay (ELISA)

Rat renal tissue (100 mg) was homogenized in 1 ml pre-cooled PBS (1 mmol/L EDTA, 0.25 mol/L sucrose, and 0.25 mmol/L PMSF) and then centrifuged at 12,000 × g for 30 min. After centrifugation, the supernatant of each sample was collected respectively. Moreover, the supernatant of cultured GMCs after different treatment was also collected respectively. The protein levels of CCL3 and CCL4 in renal tissues and GMC supernatant were examined with ELISA kits (Elabscience, China).

### Luciferase reporter assay

The plasmids of pGL3/CCL3-full-length (pGL3/CCL3-FL) and pGL3/IRF-8-FL were constructed by inserting rat CCL3 promoter (-1400 ~ +94 nt) and IRF-8 promoter (-1892 ~ +174 nt) into pGL3-basic vector (Promega, USA). We also amplified different truncated promoter fragments of CCL3 (-453 ~ +94, -352 ~ +94 and -3 ~ +94 nt) and IRF-8 (-1360 ~ +174 nt, -752 ~ +174 nt, -68 ~ +174 nt) and inserted them into the pGL3-basic. pGL3/CCL3 mutant (pGL3/CCL3-FL-M, -249 ~ -236 nt, from 5'-TCATAAGAGAAACT-3' to 5'-GACGGGTACCCGTC-3') and pGL3/IRF-8 mutant (pGL3/IRF-8-FL-M1, -591 ~ -582 nt, from 5'-GGTACTTTAC-3' to 5'-ACTCTAGAGT-3'; pGL3/IRF-8-FL-M2, -299 ~ -290 nt, from 5'-GGAAAGCACT-3' to 5'-ACTCTAGAGT-3'; pGL3/IRF-8-FL-M3 including M1 and M2 mutant) were constructed by General Biosystems. The activity of CCL3-FL and IRF-8-FL promotor, and their different truncated fragments or mutants in GMCs with different treatments was detected by luciferase reporter assay 31.

### Chromatin immunoprecipitation (ChIP)

Protein-DNA complexes were immunoprecipitated by using an antibody against IRF-8 (sc-365042) or p65 (8242), and preimmune IgG respectively 31. Within the immunoprecipitated chromatin, a proximal region in CCL3 promotor (-266 ~ -75nt) or IRF-8 promotor (-615 ~ -478 nt or -394 ~ -261 nt) was amplified by PCR using different primers (CCL3 promotor: sense, 5'-CCTACCCCACCTCTCGC-3' and antisence, 5'-GGGGCTGTGTAGGGAAAA-3'; IRF-8 promotor region 1: sense, 5'- GACACAGGCTCTGACGCA -3' and antisence, 5'- GGGTTCTGTTCCCGCTG -3'; IRF-8 promotor region 2: sense, 5'- GTGGTGCTGGGGTCAAAGT -3' and antisence, 5'- GCACCTGCTGGGAAGAAG -3').

### Lentiviral (LV) shRNA packing and *in vivo* experiments

LV-shPKC-α, LV-shp65, LV-shIRF-8, LV-shCCL3, LV-shCCL4 and LV-shCTR were provided from GenePharma. The *in vivo* shRNA sequences to silence PKC, p65 and IRF-8 genes as well as shCTR were the same as the sequences used *in vitro*. The shRNA sequences against CCL3 and CCL4 were 5'-TCACTGAGCTGGAACTAAA-3' and 5'-GGAACTTTGTGATGGATTA-3'. The male SD rats were then divided into 6 groups (n=5 in each time point/group), namely, (1) LV-shCTR + NRS (0.75ml/100g, i.v., NRS), (2) LV-shCTR + Thy-1N (0.75ml/100g, i.v., Thy-1 Ab), (3) LV-shPKC + Thy-1N, (4) LV-shp65 + Thy-1N, (5) LV-shIRF-8 + Thy-1N, (6) LV-shCCL3/4 + Thy-1N. Here, the *in vivo* experiments were done as described previously 32. The renal cortexes of rats after different treatments were collected by sacrifice at fixed time. The efficiency of LV infection into the kidney was determined by observing GFP expression (Figure S2).

### Proliferative change examination

The renal sections (4 μm) of rat cortex were stained with hematoxylin and eosin (H&E), and the mean number of total glomerular cells was counted from 100 glomerular cross-sections of each rat under light microscopy (LM). In addition, the ultrathin sections of renal cortex were stained, and the glomerular ultrastructural changes were observed under electron microscopy (EM) 26, 33.

### Immunohistochemistry (IHC) staining

Paraffin-embedded renal sections of Thy-1N rats and MsPGN patients were deparaffinized in xylol and dehydrated in ethanol. Heat-induced tissue antigen retrieval in sections was performed using citrate buffer, and then endogenous peroxidase activity in tissues was blocked with 3% hydrogen peroxide. Next, non-specific antibody binding sites in tissues were blocked with 5% normal goat serum in PBS. The rat renal tissue sections were incubated with primary antibody against CCL3 (A7568), CCL4 (A1671), CD68 (ab31630), p-PKC-α (Thr638, ab32502), p-p65 (Ser536, sc-136548) and then incubated with HRP-conjugated anti-mouse IgG (BS12478). Some of these antibodies (A7568, A1671, ab32502) were conjugated with biotin by using a biotin conjugation kit (Abcam, ab201795) according to the instructions. The patient renal tissue sections were incubated with antibodies against p-PKC-α (ab32502), p-p65 (3033), IRF-8 (sc-365042), CCL3 (A7568), CCL4 (A1671) and CD68 (ab955), and then incubated with HRP-conjugated anti-rabbit IgG (BS13278) or anti-mouse IgG (BS12478). DAB staining was done, and the quantitative analysis of positive cell number or area was performed.

### Statistical analysis

Data are presented as means ± SE. T-Test or One-way ANOVA followed by Bonferroni post-hoc test was used to determine significant differences among groups. The Pearson correlation analysis was also performed to investigate the correlation between two factors.* p*<0.05 was considered significant.

## Results

### CCL3/4 production and Mφ number are increased both in the renal tissues of Thy-1N rats and in the GMCs stimulated with sublytic C5b-9

**Figure 1:**
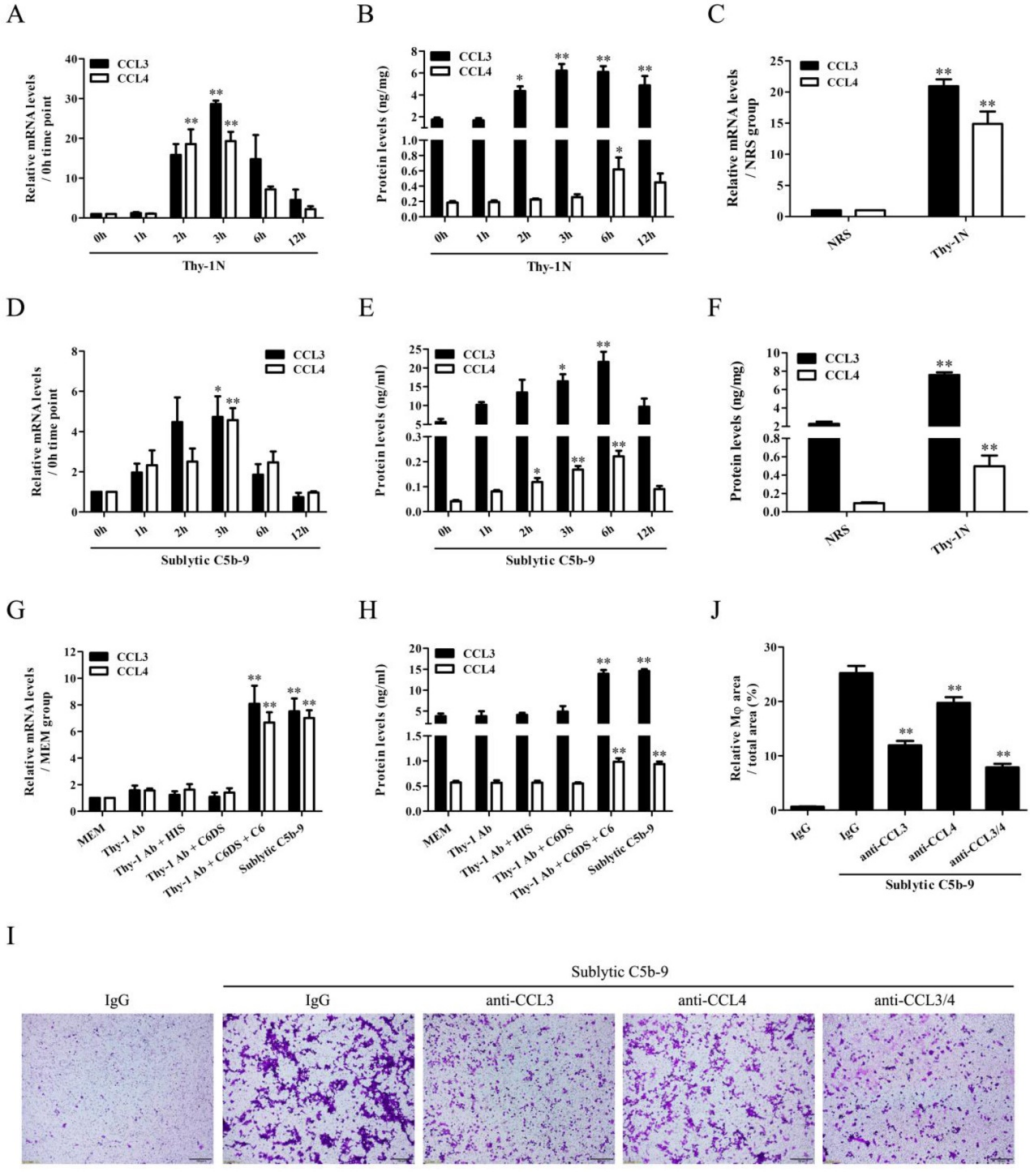
** CCL3/4 production both *in vivo* and* in vitro* as well as the effects of CCL3/4 on Mφ chemotaxis *in vitro*.** (A, B, D, E) The mRNA and protein of CCL3/4 in the renal tissues of Thy-1N rats (A and B) and in the GMCs exposed to sublytic C5b-9 (D and E) at different time points was examined by qPCR (A and D) and ELISA (B and E). * P<0.05, ** P<0.01 vs. 0 h. (C and F) CCL3/4 expression in the renal tissues of Thy-1N and NRS rats was detected by qPCR (C, at 3 h for mRNA) and ELISA (F, at 6 h for protein). ** P<0.01 vs. NRS. (G and H) Rat GMCs were divided into different groups, and then CCL3/4 was determined by qPCR (G, at 3 h for mRNA) and ELISA (H, at 6 h for protein). ** P<0.01 vs. other groups. (I and J) An upper chamber containing Mφ was separated by a membrane from a lower chamber containing GMCs following sublytic C5b-9 treatment with or without neutralizing antibodies against CCL3/4. Chemotaxis of Mφ from the upper chamber into the lower chamber was observed by the crystal violet staining of transwell membrane (Magnification: ×100). ** P<0.01 vs. IgG + sublytic C5b-9. Data were represented as means ± SE (n=3-5).

We found that CCL3/4 expression elevated in a time-dependent manner, peaked at 3h for mRNA and 6h for protein both in the renal tissues of Thy-1N rats and in the GMCs exposed to sublytic C5b-9 (Figure 1A, B, D, E). Further *in vivo* grouping experiments showed that CCL3/4 levels in the renal tissues of Thy-1N rats were higher than that of NRS-treated control rats at 3h for mRNA and 6h for protein (Figure 1C and F, Figure S3). To make sure CCL3/4 production was due to C5b-9 assembly, grouping experiments were performed, and the data displayed that CCL3/4 mRNA (3h) and protein (6h) were markedly increased in sublytic C5b-9 and Thy-1 Ab + C6DS + C6 group (Figure 1G and H), confirming that CCL3/4 gene expression is triggered by sublytic C5b-9.

Next, we continued to observe the Mφ accumulation in the renal tissues of Thy-1N rats, and the results showed that the number of Mφ (CD68-positive cells) in the glomeruli was markedly increased at 12h, more obvious at 24h (Figure S4A). In addition, transwell experiments *in vitro* exhibited that sublytic C5b-9-treated GMCs at 12h notably caused Mφ chemotaxis, and more obvious at 24h (Figure S4B). Grouping experiments also showed that Mφ increase in the renal tissues of Thy-1N rats (Figure S4C) and Mφ chemotaxis to the GMCs stimulated with sublytic C5b-9 or Thy-1 Ab + C6DS + C6 were more than that of other groups (Figure S4D). To verify whether CCL3/4 secretion from the GMCs is related to Mφ chemotaxis, the neutralizing antibodies (Abs) against CCL3/4 were used, and the results exhibited that anti-CCL3/4 Abs could reduce Mφ chemotaxis, and their combination obtained more obvious effect (Figure 1I and J). Mφ were incubated with the supernatant of GMCs induced by sublytic C5b-9, and the expression of CCR1/5 and secretion of CCL3/4 were determined. The results showed that the GMC supernatant did not significantly affect the production of CCR1/5 (Figure S5A) and CCL3/4 (Figure S5B).

### IRF-8 is up-regulated and contributes to CCL3/4 expression in the GMCs exposed to sublytic C5b-9

**Figure 2:**
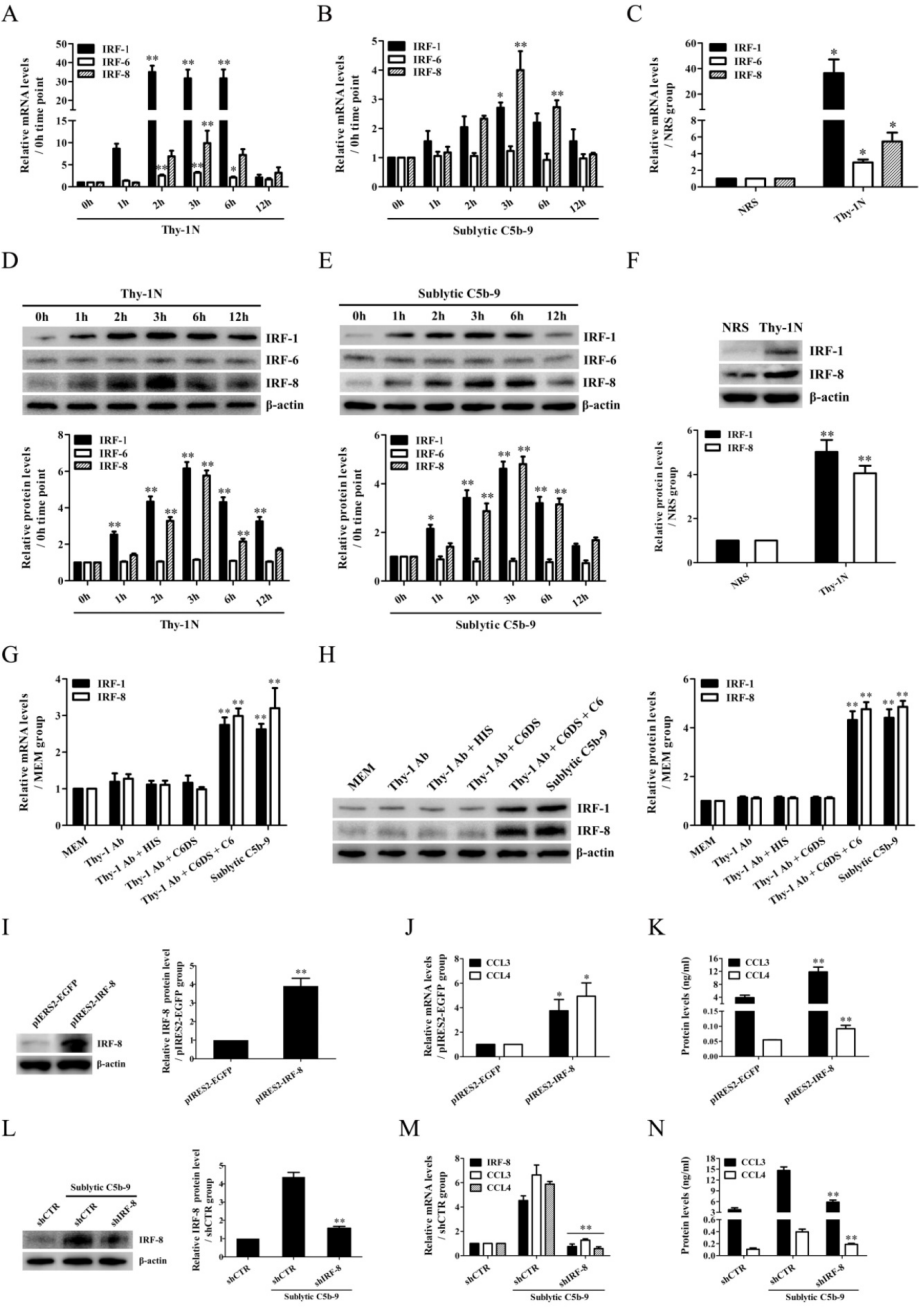
** IRF-1/6/8 expression both* in vivo* and* in vitro* as well as the roles of IRF-8 in CCL3/4 production *in vitro*.** (A, B, D, E) The expression of IRF-1/6/8 in the renal tissues of Thy-1N rats (A and D) and in the GMCs exposed to sublytic C5b-9 (B and E) at different time points was examined by qPCR (A and B) and IB (D and E). * P<0.05, ** P<0.01 vs. 0 h. (C and F) The expression of IRF-1/6/8 in the renal tissues of Thy-1N and NRS rats at 3 h was detected by qPCR (C) and IB (F). * P<0.05, ** P<0.01 vs. NRS. (G and H) Rat GMCs were divided into different groups. IRF-1/6/8 expression was determined by qPCR (G) and IB (H) at 3 h after treatment. ** P<0.01 vs. other groups. (I-K) Rat GMCs were transfected with pIRES2-IRF-8 or pIRES2-EGFP, and then the expression of IRF-8 and CCL3/4 was examined by IB (I), qPCR (J) and ELISA (K) at 48 h after transfection. * P<0.05, ** P<0.01 vs. pIRES2-EGFP. (L-N) Rat GMCs were transfected with shIRF-8 or shCTR for 48 h followed by sublytic C5b-9 treatment for 3 h. The expression of IRF-8 and CCL3/4 was detected by IB (L), qPCR (M) and ELISA (N). ** P<0.01 vs. shCTR + sublytic C5b-9. Data were represented as means ± SE (n=3-5).

In order to reveal the transcriptional regulation of CCL3/4 genes in the GMCs upon sublytic C5b-9 stimulation of Thy-1N rats, IRF family transcriptional factors including IRF-1/6/7/8 were examined. The time course and grouping experiments exhibited that the mRNA and protein of IRF-1/8 were increased in a time-dependent manner, with a maximum at 3h both *in vivo* and* in vitro* (Figure 2A-H, Figure S6). Although IRF-6 mRNA was also increased, its protein expression remained unchanged (Figure 2A-E). In addition, IRF-7 mRNA expression in the GMCs was not affected by sublytic C5b-9 stimulation (Figure S7). Therefore, we further explored the roles of IRF-1/8 in CCL3/4 expression, and found that IRF-8 overexpression notably increased CCL3/4 production (Figure 2I-K), but IRF-1 overexpression had no significant effect on CCL3/4 (Figure S8). Moreover, IRF-8 knockdown markedly reduced CCL3/4 level in rat GMCs exposed to sublytic C5b-9 (Figure 2L-N), indicating that IRF-8 is an upstream regulator of CCL3/4 expression in the GMCs stimulated with sublytic C5b-9. We also evaluated the regulatory role of IRF-8 in GMC proliferation, and the results showed that overexpression of IRF-8 in the GMCs did not significantly affect cellular proliferation (Figure S9).

### CCL3 gene transcription is mediated by IRF-8 in the GMCs upon sublytic C5b-9

**Figure 3:**
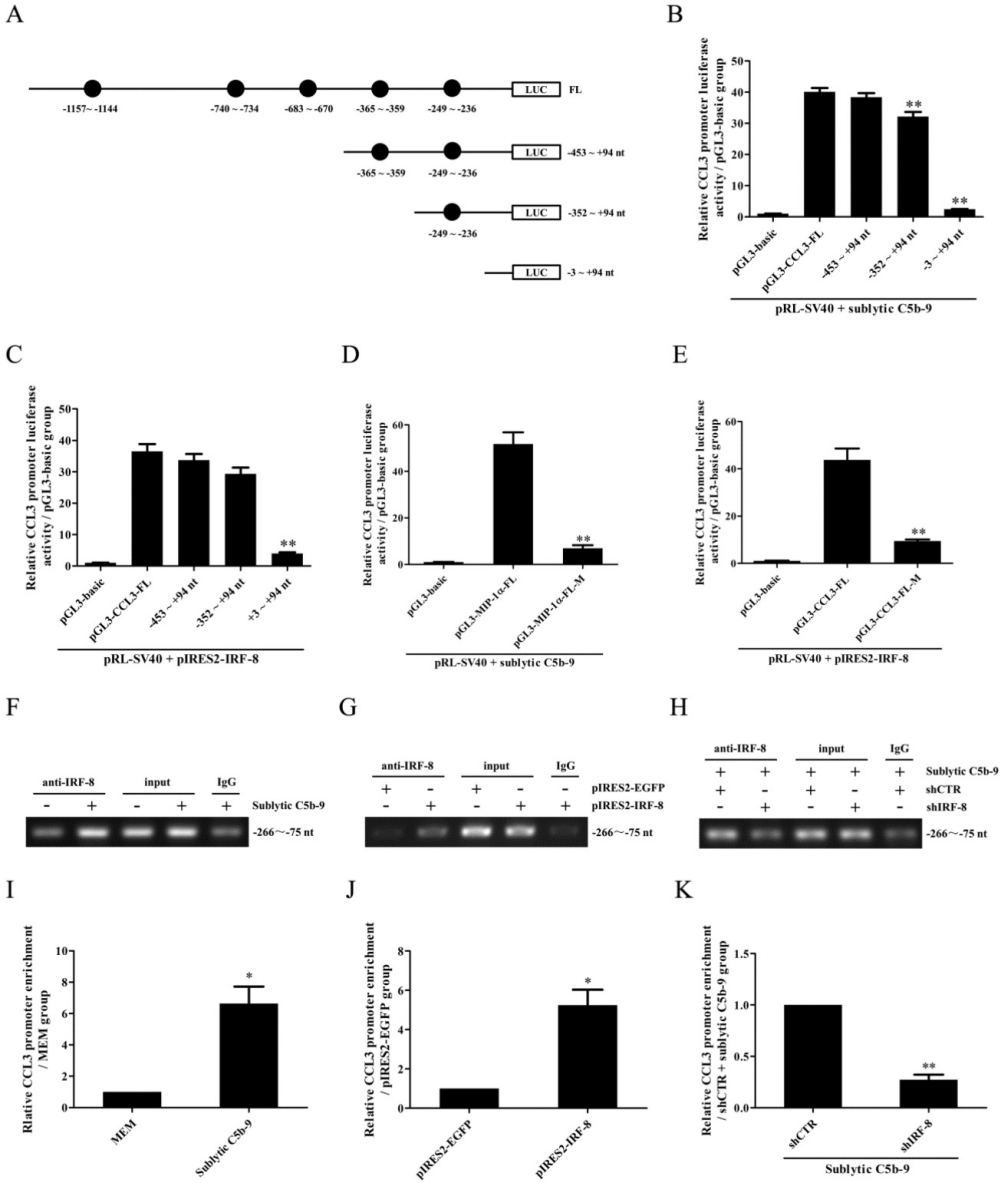
** The role of IRF-8 in CCL3 gene transcription in the GMCs upon sublytic C5b-9 stimulation.** (A) Five IRF-8-binding elements in CCL3 gene promoter were predicted with TFsearch. The FL and three truncated fragments of CCL3 promoter were constructed, including pGL3-CCL3-FL, pGL3-CCL3-1 (-453 ~ +94 nt), pGL3-CCL3-2 (-352 ~ +94 nt) and pGL3-CCL3-3 (-3 ~ +94 nt). (B) The above-mentioned plasmids and pRL-SV40 were transfected into rat GMCs in different groups for 48 h, and the luciferase activity was detected at 3 h after sublytic C5b-9 stimulation. ** P<0.01 vs. FL or first two truncated groups. (C) The plasmids of pIRES2-IRF-8, pGL3-CCL3-FL (or CCL3-1, CCL3-2, CCL3-3) and pRL-SV40 were transfected into the GMCs in different groups, and then the luciferase activity was examined at 48 h after transfection. ** P<0.01 vs. FL or first two truncated groups. (D) The plasmids of pGL3-CCL3 (FL or mutant) and pRL-SV40 were transfected into rat GMCs in different groups (for 48 h), and the luciferase activity was determined at 3 h after sublytic C5b-9 incubation. ** P<0.01 vs. FL. (E) The plasmids of pIRES2-IRF-8, pGL3-CCL3 (FL or mutant) and pRL-SV40 were transfected into the GMCs in different groups, after 48 h the luciferase activity was detected. ** P<0.01 vs. FL. (F-K) The anti-IRF-8 antibody or IgG was used to enrich DNA-IRF-8 complex in the GMCs in different groups at 48 h after plasmid transfection or at 3 h after sublytic C5b-9 treatment. Immunoprecipitated DNA was amplified by PCR (F, G, H) or qPCR (I, J, K) for CCL-3 gene promoter. * P<0.05, ** P<0.01 vs. MEM, pIRES2-EGFP or shCTR + sublytic C5b-9. Data were represented as means ± SE (n=3).

Because rat CCL4 gene promoter was not reported in NCBI Genome Search database, further experiments were done to evaluate the regulatory effect of IRF-8 on CCL3 gene promotor activity in the GMCs. Firstly, CCL3 gene promoter was analyzed with TFsearch, and according to these predicted IRF-8-binding elements (Figure 3A), the FL and three truncated fragments of CCL3 promoter were constructed, namely pGL3-CCL3-FL, pGL3-CCL3-1 (-453 ~ +94 nt), pGL3-CCL3-2 (-352 ~ +94 nt) and pGL3-CCL3-3 (-3 ~ +94 nt). Then, GMCs were transfected with pGL3-CCL3-FL/1/2/3 and pIRES2-IRF-8, followed by sublytic C5b-9 incubation or not. As shown in Figure 3B and C, sublytic C5b-9 treatment and IRF-8 overexpression obviously elevated the luciferase activity of CCL3 promotor FL and first two truncated fragments, but had no effect on the shortest fragment, suggesting that -352 ~ - 3nt of CCL3 promoter contains an effective IRF-8-binding site, i.e. the predicted IRF-8-binding site (-249 ~ -236 nt). After this binding site was mutated, sublytic C5b-9- or overexpressed IRF-8-induced promotor activity of CCL3-FL was obviously decreased (Figure 3D and E). IRF-8 knockdown reduced CCL3 gene promotor FL activity in the GMCs exposed to C5b-9 (Figure S10). Furthermore, ChIP assay demonstrated that sublytic C5b-9-induced IRF-8 or overexpressed IRF-8 could bind to -266 ~ -75 nt of CCL3 promoter (Figure 3F-K), hinting that sublytic C5b-9 can increase CCL3 promotor activity via up-regulation of IRF-8.

### PKC-α and p65 activation is required for IRF-8 expression in the GMCs in response to sublytic C5b-9

Time course and grouping experiments showed that the phosphorylation levels of ERK1/2, p38 MAPK, JNK, PKC-α, p65 and STAT3 (namely p-ERK1/2, p-p38, p-JNK, p-PKC-α, p-p65 and p-STAT3) were increased in a time-dependent manner both *in vivo* and *in vitro* (Figure S11-13). Besides, PKC-α inhibitor and p65 inhibitor could significantly reduce IRF-8 and CCL3/4 expression in the GMCs exposed to sublytic C5b-9. PKC-α inhibitor decreased PKC and p65 phosphorylation, however p65 inhibitor reduced p65 phosphorylation rather than PKC-α phosphorylation (Figure S14), suggesting that PKC-α might be an upstream regulator of p65, and PKC-α/p65 activation can up-regulate IRF-8 expression and promote CCL3/4 production.

**Figure 4:**
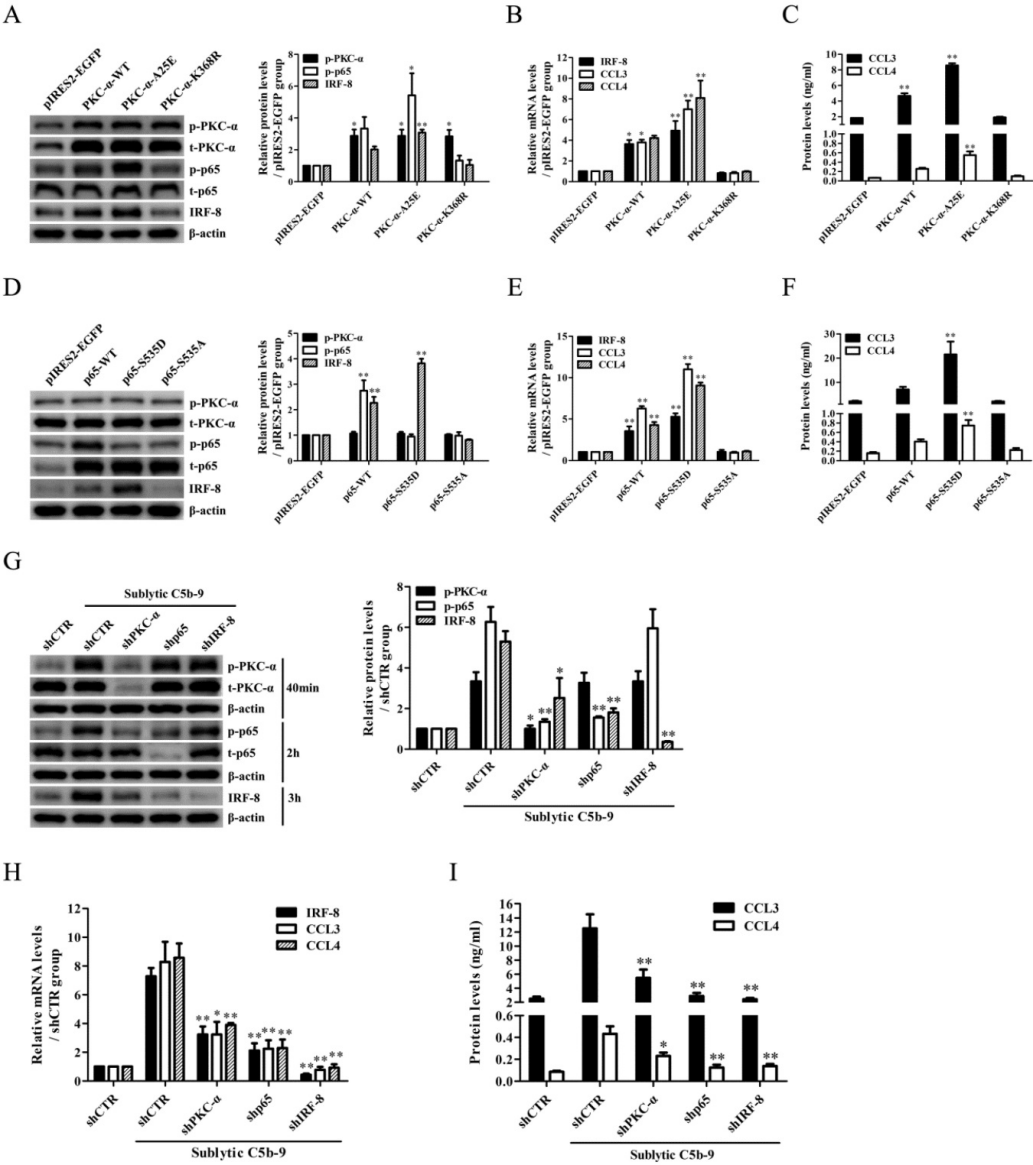
** The effects of PKC-α and p65 on IRF-8 expression in the GMCs.** (A-C) The plasmids of pIRES2-PKC-α-WT, pIRES2-PKC-α-A25E and pIRES2-PKC-α-K368R were respectively transfected into the GMCs for 48 h, and then the expression of p-PKC-α, t-PKC-α, p-p65, t-p65, IRF-8 and CCL3/4 was detected by IB (A), qPCR (B) and ELISA (C). * P<0.05, ** P<0.01 vs. pIRES2-EGFP. (D-F) The plasmids of pIRES2-PKC-α-WT, pIRES2-PKC-α-A25E or pIRES2-PKC-α-K368R were transfected into GMCs for 48 h, and then the expression of p-PKC-α, t-PKC-α, p-p65, t-p65, IRF-8 and CCL3/4 was detected by IB (D), qPCR (E) and ELISA (F). ** P<0.01 vs. pIRES2-EGFP. (G-I) Rat GMCs were transfected with shPKC-α, shp65 or shCTR for 48 h, and incubated with sublytic C5b-9 for 3 h or 6 h. The expression of IRF-8, CCL3 and CCL4 was detected by IB (G, at 3 h), qPCR (H, at 3 h) and ELISA (I, at 6 h). * P<0.05, ** P<0.01 vs. shCTR + sublytic C5b-9. Data were represented as means ± SE (n=3).

In order to confirm the roles of PKC-α and p65 in regulating IRF-8, the GMCs were transfected with wide type, active and inactive PKC-α plasmids (pIRES2-PKC-α-WT, pIRES2-PKC-α-A25E and pIRES2-PKC-α-K368R) or wide type, active and inactive p65 plasmids (pIRES2-p65-WT, pIRES2-p65-S535D and pIRES2-p65-S535A) respectively. The results showed that overexpression of wide type and active PKC-α or p65, especially active PKC-α or p65 markedly up-regulated IRF-8 and CCL3/4, and overexpression of inactive PKC-α or p65 with had no significant effect (Figure 4A-F). Similar levels of PKC-α phosphorylation at Thr638 in PKC-α-WT, PKC-α-A25E and PKC-α-K368R were detected, because the mutation of A25 and K368 did not affect the phosphorylation of Thr638. Meanwhile, knockdown of PKC-α or p65 with shPKC or shp65 obviously down-regulated IRF-8 and CCL3/4 expression induced by sublytic C5b-9 (Figure 4G-I). Notably, overexpression or knockdown of PKC-α could regulate p65 phosphorylation (Figure 4A and G), but overexpression or knockdown of p65 had no effect on PKC-α phosphorylation (Figure 4D and G), and IRF-8 overexpression did not affect PKC-α and p65 phosphorylation (Figure S15), suggesting that PKC-α/p65/IRF-8 axis might promote CCL3/4 production in the GMCs.

### IRF-8 gene induction is mediated by p65 in the GMCs stimulated by sublytic C5b-9

**Figure 5:**
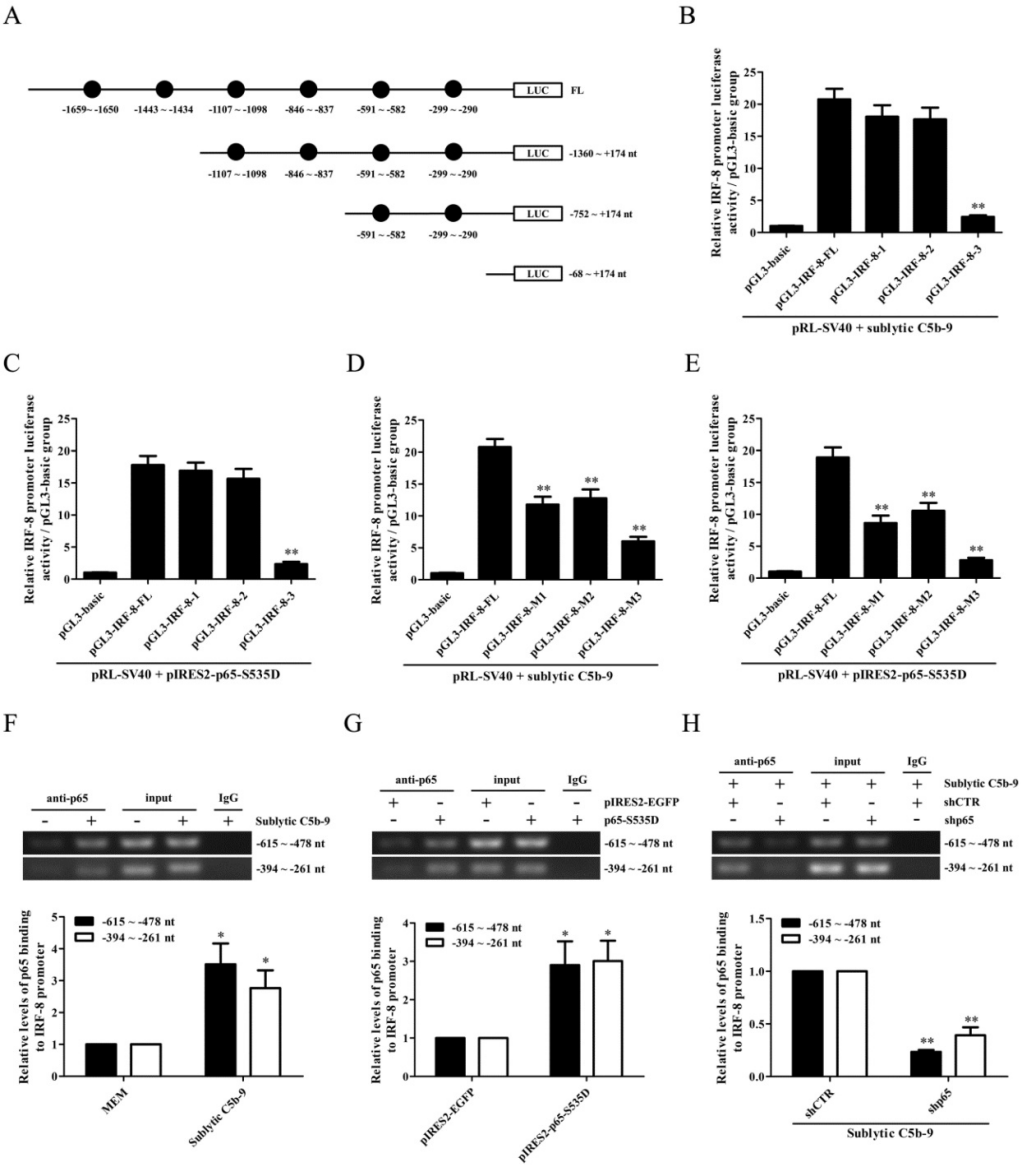
** The role of p65 in IRF-8 gene transcription in the GMCs exposed to sublytic C5b-9.** (A) According to six predicted p65-binding elements by TFsearch software, the FL and three truncated fragments of IRF-8 promoter were constructed, i.e. pGL3-IRF-8-FL, pGL3-IRF-8-1 (-1360 ~ +174 nt), pGL3-IRF-8-2 (-752 ~ +174 nt) and pGL3-IRF-8-3 (-68 ~ +174 nt). (B) The luciferase reporter plasmids of IRF-8-FL (or IRF-8-1, IRF-8-2, IRF-8-3) and pRL-SV40 were transfected into GMCs in different groups for 48 h, and then the luciferase activity was determined at 3 h after sublytic C5b-9. ** P<0.01 vs. FL or first two truncated groups. (C) The plasmids of pIRES2-p65-S535D and pGL3-IRF-8 (FL or truncated fragments) were transfected into the GMCs in different groups, after 48 h the luciferase activity was detected. ** P<0.01 vs. FL or first two deplete groups. (D) The plasmids of pGL3-IRF-8 (FL or mutant) and pRL-SV40 were transfected into GMCs in different groups for 48 h, and the luciferase activity was examined at 3 h after sublytic C5b-9 incubation. ** P<0.01 vs. FL. (E) The plasmids of pIRES2-p65-S535D, pGL3-IRF-8 (FL or mutant) and pRL-SV40 were transfected into GMCs in different groups, after 48 h the luciferase activity was measured. ** P<0.01 vs. FL. (F-H) GMCs were treated in different groups (transfection 48 h or sublytic C5b-9 stimulation 3 h), and then the anti-p65 antibody or IgG was used to purify DNA-p65 complex. Next, PCR was done to amplify IRF-8 promoter fragment from immunoprecipitated DNA. * P<0.05, ** P<0.01 vs. MEM, pIRES2-EGFP or shCTR + sublytic C5b-9. Data were represented as means ± SE (n=3).

To elucidate the regulatory role of p65 in IRF-8 gene transcription, we first constructed the FL and three truncated fragments of IRF-8 promoter, i.e. pGL3-IRF-8-FL, pGL3-IRF-8-1 (-1360 ~ +174 nt), pGL3-IRF-8-2 (-752 ~ +174 nt) and pGL3-IRF-8-3 (-68 ~ +174 nt), according to the six predicted p65-binding elements by TFsearch (Figure 5A). Then, GMCs were transfected with pGL3-IRF-8-FL/1/2/3 and pIRES2-p65-S535D, followed by sublytic C5b-9 stimulation or not. As shown in Figure 5B and C, both sublytic C5b-9 stimulation and p65-S535D overexpression greatly enhanced the luciferase activity of IRF-8 promotor FL and first two truncated fragments, but had no effect on the shortest fragment, suggesting that -752 ~ -68 nt of IRF-8 promoter contains p65-binding sites. Additionally, the knockdown of p65 reduced IRF-8 gene promotor FL activity in the GMCs exposed to C5b-9 (Figure S16). After the two predicted p65-binding sites (-591 ~ -582 nt and -299 ~ -290 nt) were mutated, the luciferase activity of IRF-8 promotor FL was markedly decreased (Figure 5D and E). Further ChIP assay revealed that sublytic C5b-9 treatment or p65 overexpression could bind to the region (-615 ~ -478 nt or -394 ~ -261 nt) of IRF-8 promoter (Figure 5F-H), implicating that sublytic C5b-9 increases IRF-8 promotor activity via up-regulation of p65. Additionally, p65 knockdown in the GMCs did not reduce CCL3 gene transcription induced by IRF-8 overexpression (Figure S17), indicating that p65 does not directly promote CCL3 gene transcription in the GMCs.

### Knockdown of renal PKC-α, p65, IRF-8 and CCL3/4 genes abolishes Mφ number, GMC proliferative changes of Thy-1N rats

**Figure 6:**
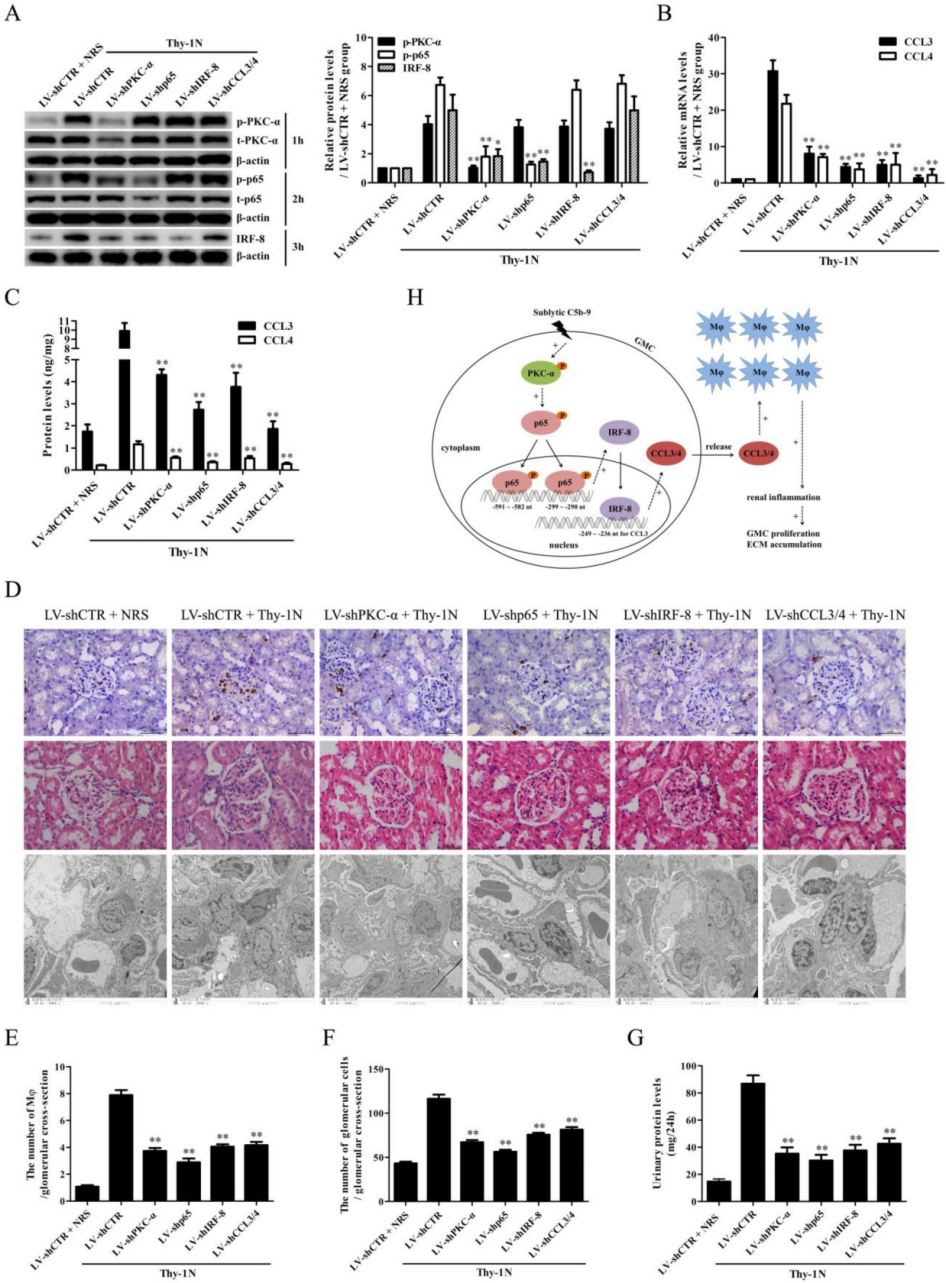
** Knockdown of renal PKC-α, p65, IRF-8 and CCL3/4 genes abolishes Mφ number, GMC proliferation, ECM accumulation and proteinuria of Thy-1N rats.** LV-shPKC-α, LV-shp65, LV-shIRF-8 and LV-shCCL3/4 were injected to silence target genes, and then nephritis was induced on 4 d after LV injection. (A-C) The levels of p-PKC-α, p-p65, IRF-8 and CCL3/4 in rat renal tissues were detected by IB (A), qPCR (B) and ELISA (C). (D-F) The numbers of CD68-positive cells in glomeruli of rats at 24 h and the numbers of total glomerular cells on 7 d were observed by IHC and H&E staining under LM (Magnification, ×400). Additionally, GMC proliferation and ECM accumulation on 7 d were evaluated by EM. (G) Rat urinary protein (mg/24h) on 7 d was detected. * P<0.05, ** P<0.01 vs. LV-shCTR + Thy-1N. Data were represented as means ± SE (n=5). (H) A schematic drawing for the pathway we revealed.

To further examine the roles of PKC-α, p65, IRF-8 and CCL3/4 in Thy-1N, the LV-shPKC-α, LV-shp65, LV-shIRF-8 and LV-shCCL3/4 were used to silence the corresponding target genes in rat renal tissues for 4 days, and then the rats in different groups were induced Thy-1N. As displayed in Figure 6, (1) PKC-α knockdown decreased p65 phosphorylation as well as IRF-8 and CCL3/4 expression. (2) P65 knockdown reduced IRF-8 and CCL3/4 expression, but had no role in PKC-α phosphorylation. (3) IRF-8 knockdown inhibited CCL3/4 production, but did not affect PKC-α and p65 phosphorylation (Figure 6A-C). (4) Knockdown of above-mentioned genes not only reduced the renal Mφ number (CD68-positive cells, Figure 6D and E), but also lessened the total glomerular cell number (Figure 6D and F), GMC proliferation and ECM accumulation (Figure 6D) as well as the content of urinary protein (mg/24h) of Thy-1N rats on 7d (Figure 6G).

### Expression of p-PKC-α, p-p65, IRF-8, CCL3/4 and accumulation of Mφ were increased in the glomeruli of MsPGN patients

**Figure 7:**
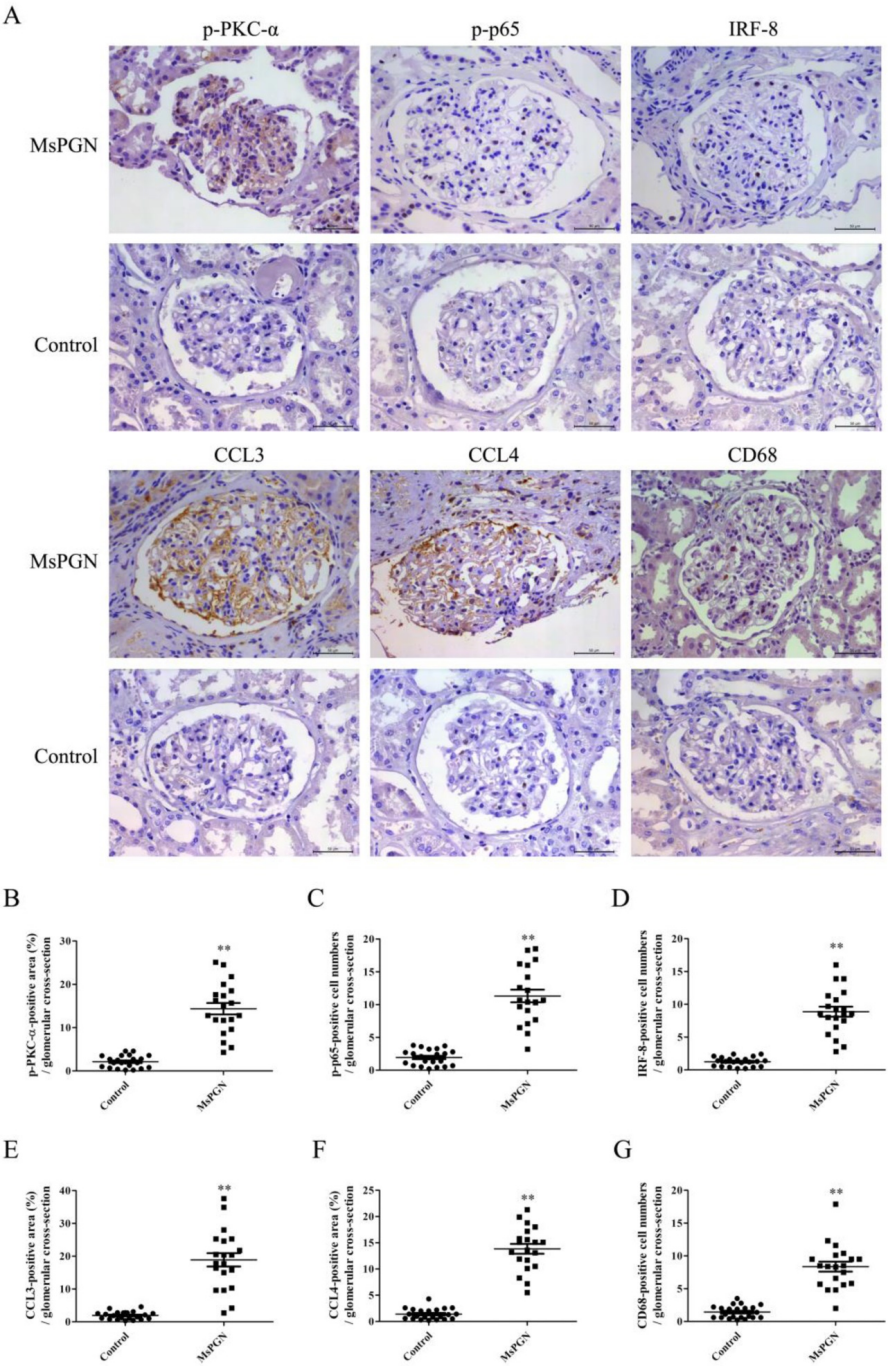
** Expression of p-PKC-α, p-p65, IRF-8, CCL3/4 and CD68 in the glomeruli of MsPGN patients.** (A) The expression of p-PKC-α, p-p65, IRF-8, CCL3/4 and CD68 in the glomeruli of MsPGN patients (n=20) and control (adjacent tissues of renal cell carcinoma patients, n=22) was determined by IHC staining (Magnification: ×200). (B-G) The quantitative analysis of the positive cell numbers or positive area was performed for p-PKC-α (B), p-p65 (C), IRF-8 (D), CCL3 (E), CCL4 (F) and CD68 (G). Data were represented as mean ± SE. ** P<0.01 vs. control.

IHC staining displayed that the levels of p-PKC-α, p-p65, IRF-8, CCL3/4 expression and Mφ accumulation in the glomeruli of MsPGN patients were not only obviously increased compared to controls (Figure 7), but also positively related with each other (Figure S18), suggesting that the elevation of p-PKC-α, p-p65, IRF-8, CCL3/4 expression and Mφ accumulation might be involved in MsPGN pathogenesis.

## Discussion

Human MsPGN is a common form of primary glomerulonephritis. Many studies have demonstrated that complement system activation (i.e. C5b-9 formation), Mφ infiltration, pro-inflammatory cytokine and chemokine production all contribute to the renal pathological changes of MsPGN 1-3, 6, 34-36. As a well-established animal model of MsPGN, Thy-1N pathogenesis is dependent on complement particularly sublytic C5b-9 8-12. Our previous studies have revealed that the production of some pro-inflammatory cytokines and chemokines including IL-6, IL-23, IL-36α, MCP-1 and RANTES was obviously increased in the renal tissues of Thy-1N rats and in the GMCs exposed to sublytic C5b-9 13-15. The current experiments found that other chemokines such as CCL3/4 was also markedly up-regulated both *in vivo* and *in vitro*, and led to Mφ chemotaxis *in vitro*. Furthermore, the concentration of CCL3/4 especially CCL3 in the GMC supernatant stimulated with sublytic C5b-9 was higher than that of MCP-1 (data not shown), suggesting that CCL3/4 might play more important roles in GMC-induced Mφ chemotaxis. These data indicate that sublytic C5b-9, as a trigger for GMC response, could induce the renal inflammatory reaction in Thy-1N rats.

Reportedly, the expression of genes is often associated with the up-regulation of certain transcription factors 37, 38. IRF is a family of transcription factors including IRF-1~9. Several IRF members e.g. IRF-1, IRF-6, IRF-7 and IRF-8 are able to increase the expression of some pro-inflammatory cytokines and chemokines, and closely related to inflammatory diseases 38-45. For example, up-regulated IRF-1 promotes liver ischemia/reperfusion injury after transplant, and the mechanism involves in elevating IL-15/IL-15Rα production in hepatocytes and accumulation of NK, NKT, and CD8^+^ T cells in liver tissues 43. In addition, the activation of IL-1 receptor-associated kinase-1 (IRAK1) or receptor-interacting protein kinase 4 (RIPK4) increases CXCL11 and RANTES gene transcription via IRF6 in the keratinocytes of oral mucosa, resulting in oral inflammation 44, 46. Furthermore, IRF-8-enhanced NLR family apoptosis inhibitory protein (NAIP) promotes NLRC4 inflammasome formation including IL-1β/IL-18 generation in the bone marrow-derived macrophages (BMDMs) of the mice infected with Salmonella typhimurium 45. However, it is still unclear about the expression of IRF-1/6/7/8 in the renal tissues of MsPGN patients. Given that IRF-1/6/7/8 have important regulatory effects on the induction of some pro-inflammatory cytokines or chemokines, our study focused on the roles of these IRFs in CCL3/4 production from the GMCs stimulated with sublytic C5b-9 in Thy-1N rats.

Our present experiments confirmed that the expression of IRF-1 and IRF-8 rather than IRF-6 and IRF-7 was increased both in the renal tissues of Thy-1N rats and in the GMCs induced by sublytic C5b-9. Thereafter, we studied the effects of IRF-1 and IRF-8 on CCL3/4 induction, and found that the increase of IRF-8 promoted CCL3/4 gene expression and protein production in the GMCs exposed to sublytic C5b-9. As for IRF-1, although our previous experiments proved that up-regulated IRF-1 could increase the expression of apoptosis-related genes including XIAP-associated factor 1 (XAF1), TNF receptor-associated death domain (TRADD) and caspase 8 in the GMCs 32, 47, the current results showed that IRF-1 up-regulation did not affect CCL3/4 expression. Next, we further identified an IRF-8-binding element (-249 ~ -236 nt) in CCL3 gene promotor and revealed that IRF-8 could strengthen CCL3 gene transcription through binding to this element. Unfortunately, because rat CCL4 gene promotor was not reported in NCBI Genome Search database, we could not study it for the time being. As we know, IRF-8 can regulate many biological behaviors such as apoptosis, proliferation, differentiation and autophagy 48-50, and meanwhile, IRF-8 can enhance the generation of some chemokines and regulate inflammatory response 16, 51. For example, interferon-gamma (IFN-γ) or lipopolysaccharide (LPS)-up-regulated IRF-8 can bind to PU.1 and NF-κB to form complexes that elevate their ability to bind to DNA through DBD regions and promote Rantes transcription and expression in mouse macrophages 16. Moreover, a high amount of IRF-8 can also boost CCL4/7/12, CXCL9/10 and Rantes generation in mouse brain tissues and drive inflammation lesions during cerebral malaria 51. Similarly, hepatic IRF-8 increase aggravates liver ischemia/reperfusion injury in mice, and the underlying mechanism is related to elevating CXCL1/9 production and neutrophil recruitment 52. Collectively, these researches indicate that IRF-8-regulated chemokine generation could play essential roles in some inflammatory diseases. In addition, IRF-8 is also involved in some other inflammatory diseases. Epidemiological studies have identified IRF-8 as a susceptibility factor for multiple sclerosis (MS) 53, 54. MS animal model experiments shows that IRF-8 enhances αvβ8 integrin expression in antigen-presenting cells (APCs) and activated TGF-β signaling leading to Th17 cell differentiation and promotes neuroinflammation of mouse experimental autoimmune encephalomyelitis (EAE) 18. Additionally, IRF8 is crucial in modulating phenotype switching and neointima formation of smooth muscle cells (SMCs) in response to vascular injury 55. These data indicate that IRF-8 might be a potential therapeutic target in some diseases.

It has been documented that the cells stimulated with external stimulants can rapidly turn on some signal transduction pathways and up-regulate some transcription factors**,** leading to corresponding target gene expression 56, 57. Subsequently, in order to find the upstream regulatory molecules of IRF-8-mediated CCL3/4 production in Thy-1N, we examined the phosphorylation (i.e. activation) of certain signal molecules and transcription factors. The results exhibited that the phosphorylation levels of ERK1/2, p38 MAPK, JNK, PKC-α, p65 and STAT3 were significantly elevated both in the renal tissues of Thy-1N rats and in the GMCs stimulated with sublytic C5b-9. Functional experiments revealed that the inhibition of PKC-α or p65 rather than ERK1/2, p38 MAPK, JNK and STAT3 could markedly down-regulate IRF-8 and CCL3/4 expression in the GMCs upon sublytic C5b-9 treatment. Therefore, PKC-α and NF-κB-p65 were chosen for continued study. Overexpression and small interference assays showed that there was an upstream and downstream relationship between PKC-α, p65 and IRF-8 during CCL3/4 induction, namely PKC-α-mediated p65 activation contributed to IRF-8-mediated CCL3/4 expression. Mechanically, as a transcription factor, p65 could bind to two binding sites (-591 ~ -582 nt and -299 ~ -290 nt) of IRF-8 gene promotor and increase IRF-8 gene transcription. Here, it is worthy to mention that, since reportedly p65 triggers the transcription of some pro-inflammatory genes such as IL-6, TNF-α and CXCL5 56, 57, and serval p65-binding sites are predicted in CCL3 gene promotor, we speculate that p65 could activate CCL3 gene in rat GMCs directly. Thus, we did further experiments, and found that p65 knockdown in the GMCs did not reduce CCL3 gene transcription induced by IRF-8 overexpression, suggesting that p65 does not directly trigger CCL3 gene transcription in the GMCs.

Our studies* in vitro* discovered that the mechanism of sublytic C5b-9-induced CCL3/4 production from the GMCs involved in the activation of PKC-α/p65/IRF-8 pathway, and contributed to the Mφ chemotaxis. To further confirm the role of above-mentioned pathway, the rat renal gene knockdown experiments were done by renal artery perfusion of different LV-shRNA in advance. The results *in vivo* displayed that PKC-α silencing decreased the levels of p-p65, IRF-8 and CCL3/4, and p65 silencing reduced the expression of IRF-8 and CCL3/4 but not p-PKC-α, additionally IRF-8 silencing inhibited the production of CCL3/4 rather than p-PKC-α and p-p65. Moreover, knockdown of renal PKC-α, p65, IRF-8 and CCL3/4 genes respectively could markedly abolished Mφ number, GMC proliferative changes and urinary protein production of Thy-1N rats. These data indicate that PKC-α/p65/IRF-8 axis could promote renal CCL3/4 expression, Mφ increase and tissue damage of Thy-1N rats (Figure 6H). Meantime, we also observed that the expression of p-PKC-α, p-p65, IRF-8, CCL3/4 proteins and the accumulation of Mφ in the renal tissues of MsPGN patients not only markedly increased, but also showed positive relationship. Taken together, our findings might provide a novel insight into the pathogenesis of renal inflammatory lesions of Thy-1N rats and MsPGN patients, and suggest some potential therapeutic targets in human MsPGN, such as the pharmacological inhibitors to PKC-α and p65, or neutralizing antibodies against CCL3 and CCL4.

## Supplementary Material

Supplementary figures and table.

## Abbreviations

CCK-8: cell counting kit-8
ChIP: chromatin immunoprecipitation
C6DS: C6-deficient serum
DMEM: Dulbecco modified Eagle's medium
ECM: extracellular matrix
ELISA: enzyme-linked immunosorbent assay
EM: electron microscopy
FBS: fetal bovine serum
GMC: glomerular mesangial cell
H&E: hematoxylin and eosin
HIS: heat-inactivated human serum
HRP: horseradish peroxidase
IB: immunoblotting
IHC: immunohistochemistry
IL: interleukin
IRF: interferon regulatory factor
LM: light microscopy
LV: lentiviral
MAPK: mitogen-activated protein kinase
Mφ: macrophage
MsPGN: mesangioproliferative glomerulonephritis
NF-κB-p65: nuclear factor kappa-B-p65
NHS: normal human sera
PCR: polymerase chain reaction
PKC-α: protein kinase C-alpha
qPCR: quantitative real-time PCR
RT-PCR: reverse transcription-PCR
SD: Sprague-Dawley
shCTR: control scrambled shRNA
Thy-1 Ab: Thy-1 antigen
Thy-1N: Thy-1 nephritis
WT: wide type

## References

[1] Soares MF, Genitsch V, Chakera A, Smith A, MacEwen C, Bellur SS (2019). Relationship between renal CD68(+) infiltrates and the Oxford Classification of IgA nephropathy. Histopathology.

[2] Wu CY, Hua KF, Hsu WH, Suzuki Y, Chu LJ, Lee YC (2020). IgA Nephropathy Benefits from Compound K Treatment by Inhibiting NF-kappaB/NLRP3 Inflammasome and Enhancing Autophagy and SIRT1. J Immunol.

[3] Xie MX, Wu ZJ, Ying S, Liu LF, Zhao CH, Yao CL (2021). Sublytic C5b-9 induces glomerular mesangial cell proliferation via ERK1/2-dependent SOX9 phosphorylation and acetylation by enhancing Cyclin D1 in rat Thy-1 nephritis. Exp Mol Med.

[4] Peng ZG, Tian J, Cui XQ, Xian WH, Sun HB, Li EG (2013). Increased number of Th22 cells and correlation with Th17 cells in peripheral blood of patients with IgA nephropathy. Hum Immunol.

[5] Li GH, Wu W, Zhang XY, Huang Y, Wen YB, Li XM (2018). Serum levels of tumor necrosis factor alpha in patients with IgA nephropathy are closely associated with disease severity. BMC Nephrol.

[6] Stangou M, Alexopoulos E, Pantzaki A, Leonstini M, Memmos D (2008). C5b-9 glomerular deposition and tubular alpha3beta1-integrin expression are implicated in the development of chronic lesions and predict renal function outcome in immunoglobulin A nephropathy. Scand J Urol Nephrol.

[7] Turnberg D, Cook HT (2005). Complement and glomerulonephritis: new insights. Curr Opin Nephrol Hypertens.

[8] Liu SM, Huang ZM, Tang A, Wu XQ, Aube J, Xu L (2020). Inhibition of RNA-binding protein HuR reduces glomerulosclerosis in experimental nephritis. Clin Sci (Lond).

[9] Wu D, Bai JX, Cui SY, Fu B, Yin ZW, Cai GY (2020). Renal progenitor cells modulated by angiotensin II receptor blocker (ARB) medication and differentiation towards podocytes in anti-thy1.1 nephritis. Ann Transl Med.

[10] Lu T, Bian Y, Zhu Y, Guo MJ, Yang Y, Guo JM (2020). HUANGKUISIWUFANG inhibits pyruvate dehydrogenase to improve glomerular injury in anti-Thy1 nephritis model. J Ethnopharmacol.

[11] Qiu W, Che N, Feng XF, Xia M, Wang H, Zhao D (2009). Apoptosis of glomerular mesangial cells induced by sublytic C5b-9 complexes in rats with Thy-1 nephritis is dependent on Gadd45 gamma upregulation. Eur J Immunol.

[12] Sato T, Van Dixhoorn MG, Prins FA, Mooney A, Verhagen N, Muizert Y (1999). The terminal sequence of complement plays an essential role in antibody-mediated renal cell apoptosis. J Am Soc Nephrol.

[13] Zhang J, Xie MX, Xia L, Yu TY, He FX, Zhao CH (2018). Sublytic C5b-9 Induces IL-23 and IL-36a Production by Glomerular Mesangial Cells via PCAF-Mediated KLF4 Acetylation in Rat Thy-1 Nephritis. J Immunol.

[14] Zhang J, Li Y, Shan K, Wang LL, Qiu W, Lu YL (2014). Sublytic C5b-9 induces IL-6 and TGF-beta1 production by glomerular mesangial cells in rat Thy-1 nephritis through p300-mediated C/EBPbeta acetylation. Faseb J.

[15] Yu TY, Gong YJ, Liu Y, Xia L, Zhao CH, Liu LF (2020). KLF6 Acetylation Promotes Sublytic C5b-9-Induced Production of MCP-1 and RANTES in Experimental Mesangial Proliferative Glomerulonephritis. Int J Biol Sci.

[16] Liu JG, Ma XJ (2006). Interferon regulatory factor 8 regulates RANTES gene transcription in cooperation with interferon regulatory factor-1, NF-kappaB, and PU.1. J Biol Chem.

[17] Nardi V, Naveiras O, Azam M, Daley GQ (2009). ICSBP-mediated immune protection against BCR-ABL-induced leukemia requires the CCL6 and CCL9 chemokines. Blood.

[18] Yoshida Y, Yoshimi R, Yoshii H, Kim D, Dey A, Xiong H (2014). The transcription factor IRF8 activates integrin-mediated TGF-beta signaling and promotes neuroinflammation. Immunity.

[19] Sukumaran P, Sun YY, Antonson N, Singh BB (2018). Dopaminergic neurotoxins induce cell death by attenuating NF-kappaB-mediated regulation of TRPC1 expression and autophagy. FASEB J.

[20] Panicker N, Sarkar S, Harischandra DS, Neal M, Kam TI, Jin H (2019). Fyn kinase regulates misfolded alpha-synuclein uptake and NLRP3 inflammasome activation in microglia. J Exp Med.

[21] Meisel M, Hermann-Kleiter N, Hinterleitner R, Gruber T, Wachowicz K, Pfeifhofer-Obermair C (2013). The kinase PKCalpha selectively upregulates interleukin-17A during Th17 cell immune responses. Immunity.

[22] Zou J, Chen ZY, Wei XB, Chen ZG, Fu YM, Yang XY (2017). Cystatin C as a potential therapeutic mediator against Parkinson's disease via VEGF-induced angiogenesis and enhanced neuronal autophagy in neurovascular units. Cell Death Dis.

[23] Rosse C, Linch M, Kermorgant S, Cameron AJ, Boeckeler K, Parker PJ (2010). PKC and the control of localized signal dynamics. Nat Rev Mol Cell Biol.

[24] Feist M, Schwarzfischer P, Heinrich P, Sun XN, Kemper J, von Bonin F (2018). Cooperative STAT/NF-kappaB signaling regulates lymphoma metabolic reprogramming and aberrant GOT2 expression. Nat Commun.

[25] Gao Y, Xiao XS, Zhang CL, Yu WD, Guo W, Zhang ZF (2017). Melatonin synergizes the chemotherapeutic effect of 5-fluorouracil in colon cancer by suppressing PI3K/AKT and NF-kappaB/iNOS signaling pathways. J Pineal Res.

[26] Xu JH, Qiu W, Wang YW, Xu J, Tong JX, Gao LJ (2006). Gene expression profile and overexpression of apoptosis-related genes (NGFI-B and Gadd 45 gamma) in early phase of Thy-1 nephritis model. Cell Tissue Res.

[27] Wang YW, He QZ, Qin HL, Xu JH, Tong JX, Gao LJ (2006). The complement C5b-9 complexes induced injury of glomerular mesangial cells in rats with Thy-1 nephritis by increasing nitric oxide synthesis. Life Sci.

[28] Gao LJ, Qiu W, Wang YW, Xu WH, Xu J, Tong JX (2006). Sublytic complement C5b-9 complexes induce thrombospondin-1 production in rat glomerular mesangial cells via PI3-k/Akt: association with activation of latent transforming growth factor-beta1. Clin Exp Immunol.

[29] Gao LJ, Zhang Y, Qiu W, Xu WH, Feng XF, Ren J (2009). Effects of PI3-k/Akt short hairpin RNA on proliferation, fibronectin production and synthesis of thrombospondin-1 and transforming growth factor-beta1 in glomerular mesangial cells induced by sublytic C5b-9 complexes. Cell Prolif.

[30] Qiu W, Zhang Y, Liu XM, Zhou JB, Li Y, Zhou Y (2012). Sublytic C5b-9 complexes induce proliferative changes of glomerular mesangial cells in rat Thy-1 nephritis through TRAF6-mediated PI3K-dependent Akt1 activation. J Pathol.

[31] Liu LS, Qiu W, Wang H, Li Y, Zhou JB, Xia M (2012). Sublytic C5b-9 complexes induce apoptosis of glomerular mesangial cells in rats with Thy-1 nephritis through role of interferon regulatory factor-1-dependent caspase 8 activation. J Biol Chem.

[32] Qiu W, Zhou JB, Zhu GQ, Zhao D, He FX, Zhang J (2014). Sublytic C5b-9 triggers glomerular mesangial cell apoptosis via XAF1 gene activation mediated by p300-dependent IRF-1 acetylation. Cell Death Dis.

[33] Plank C, Hartner A, Klanke B, Geissler B, Porst M, Amann K (2005). Adrenomedullin reduces mesangial cell number and glomerular inflammation in experimental mesangioproliferative glomerulonephritis. Kidney Int.

[34] Gan L, Li XZ, Zhu MY, Chen C, Luo HM, Zhou QL (2018). Acteoside relieves mesangial cell injury by regulating Th22 cell chemotaxis and proliferation in IgA nephropathy. Ren Fail.

[35] Maekawa K, Shibano T, Sawaki J, Mae H, Hattori M, Tanizawa T (2014). Clinical usefulness of CD68 staining in children with various glomerular diseases. Nihon Jinzo Gakkai Shi.

[36] Segarra-Medrano A, Carnicer-Caceres C, Valtierra-Carmeno N, Agraz-Pamplona I, Ramos-Terrades N, Jatem Escalante E (2017). Study of the variables associated with local complement activation in IgA nephropathy. Nefrologia.

[37] Li JJ, Liao TT, Liu HY, Yuan HL, Ouyang TH, Wang JJ (2021). Hypoxic Glioma Stem Cell-Derived Exosomes Containing Linc01060 Promote Progression of Glioma by Regulating the MZF1/c-Myc/HIF1alpha Axis. Cancer Res.

[38] Irving AT, Zhang Q, Kong PS, Luko K, Rozario P, Wen M (2020). Interferon Regulatory Factors IRF1 and IRF7 Directly Regulate Gene Expression in Bats in Response to Viral Infection. Cell Rep.

[39] Karki R, Lee E, Sharma BR, Banoth B, Kanneganti TD (2020). IRF8 Regulates Gram-Negative Bacteria-Mediated NLRP3 Inflammasome Activation and Cell Death. J Immunol.

[40] Kowalec K, Wright GEB, Drogemoller BI, Aminkeng F, Bhavsar AP, Kingwell E (2018). Common variation near IRF6 is associated with IFN-beta-induced liver injury in multiple sclerosis. Nat Genet.

[41] Shen YB, Sun ZF, Mao SR, Zhang YN, Jiang WL, Wang HT (2020). IRF-1 contributes to the pathological phenotype of VSMCs during atherogenesis by increasing CCL19 transcription. Aging.

[42] Huynh J, Scholz GM, Aw J, Kwa MQ, Achuthan A, Hamilton JA (2016). IRF6 Regulates the Expression of IL-36gamma by Human Oral Epithelial Cells in Response to Porphyromonas gingivalis. J Immunol.

[43] Yokota S, Yoshida O, Dou L, Spadaro AV, Isse K, Ross MA (2015). IRF-1 promotes liver transplant ischemia/reperfusion injury via hepatocyte IL-15/IL-15Ralpha production. J Immunol.

[44] Kwa MQ, Scholz GM, Reynolds EC (2016). RIPK4 activates an IRF6-mediated proinflammatory cytokine response in keratinocytes. Cytokine.

[45] Dong XC, Hu XM, Bao Y, Li G, Yang XD, Slauch JM (2021). Brd4 regulates NLRC4 inflammasome activation by facilitating IRF8-mediated transcription of Naips. J Cell Biol.

[46] Kwa MQ, Nguyen T, Huynh J, Ramnath D, De Nardo D, Lam PY (2014). Interferon regulatory factor 6 differentially regulates Toll-like receptor 2-dependent chemokine gene expression in epithelial cells. J Biol Chem.

[47] Zhu GQ, Qiu W, Li YT, Zhao CH, He FX, Zhou MY (2017). Sublytic C5b-9 Induces Glomerular Mesangial Cell Apoptosis through the Cascade Pathway of MEKK2-p38 MAPK-IRF-1-TRADD-Caspase 8 in Rat Thy-1 Nephritis. J Immunol.

[48] Masuda T, Tsuda M, Yoshinaga R, Tozaki-Saitoh H, Ozato K, Tamura T (2012). IRF8 is a critical transcription factor for transforming microglia into a reactive phenotype. Cell Rep.

[49] Sichien D, Scott CL, Martens L, Vanderkerken M, Van Gassen S, Plantinga M (2016). IRF8 Transcription Factor Controls Survival and Function of Terminally Differentiated Conventional and Plasmacytoid Dendritic Cells, Respectively. Immunity.

[50] Gupta M, Shin DM, Ramakrishna L, Goussetis DJ, Platanias LC, Xiong HB (2015). IRF8 directs stress-induced autophagy in macrophages and promotes clearance of Listeria monocytogenes. Nat Commun.

[51] Berghout J, Langlais D, Radovanovic I, Tam M, MacMicking JD, Stevenson MM (2013). Irf8-regulated genomic responses drive pathological inflammation during cerebral malaria. PLoS Pathog.

[52] Shi GJ, Zhang ZX, Ma SQ, Li Y, Du SJ, Chu Y Hepatic interferon regulatory factor 8 expression mediates liver ischemia/reperfusion injury in mice. Biochem Pharmacol. 2021: 192: 114728.

[53] De Jager PL, Jia XM, Wang J, de Bakker PI, Ottoboni L, Aggarwal NT (2009). Meta-analysis of genome scans and replication identify CD6, IRF8 and TNFRSF1A as new multiple sclerosis susceptibility loci. Nat Genet.

[54] Chrabot BS, Kariuki SN, Zervou MI, Feng X, Arrington J, Jolly M (2013). Genetic variation near IRF8 is associated with serologic and cytokine profiles in systemic lupus erythematosus and multiple sclerosis. Genes Immun.

[55] Zhang SM, Gao L, Zhang XF, Zhang R, Zhu LH, Wang PX (2014). Interferon regulatory factor 8 modulates phenotypic switching of smooth muscle cells by regulating the activity of myocardin. Mol Cell Biol.

[56] Lunazzi G, Buxade M, Riera-Borrull M, Higuera L, Bonnin S, Huerga Encabo H (2021). NFAT5 Amplifies Antipathogen Responses by Enhancing Chromatin Accessibility, H3K27 Demethylation, and Transcription Factor Recruitment. J Immunol.

[57] Tsui KH, Chang KS, Sung HC, Hsu SY, Lin YH, Hou CP (2021). Mucosa-Associated Lymphoid Tissue 1 Is an Oncogene Inducing Cell Proliferation, Invasion, and Tumor Growth via the Upregulation of NF-kappaB Activity in Human Prostate Carcinoma Cells. Biomedicines.

