# Supplementary material for: Sublytic C5b-9 Induces CCL3/4 Production and Macrophage Accumulation in Thy-1N Rats via PKC-α/p65/IRF-8 Axis
Source: Int J Biol Sci. 2022 May 1;18(8):3178–93. doi: 10.7150/ijbs.69652 (PMC9134925; doi:10.7150/ijbs.69652)
Supplement: Supplementary file 1 — Supplementary figures and table. [file ijbsv18p3178s1.pdf]

Table S1

	forward primer (5'→3')	reverse primer (5'→3')
CCL3	GCAGCGAGTACCAGTCCCTT	ACTTCGTTCCAGAGCGCCAT
CCL4	CTGCCTTCTCTCTCCTCCTG	AGGGTCAGAGCCTATTGGTG
IRF-1	TCTCTGATCAACACCTCTCAGCA	CATACTTGCTAAGGTTCTGTCCAG
IRF-6	ATCTCTGATCATCTCAGCACTACA	TCTGTCCAGCATACTTGCTAAGGT
IRF-7	TCTGGATGAAGCTGATGCAC	AGAGCTGGGCCAGTTGTAGA
IRF-8	AGCAGGGTGGTTCTGTGCTT	GCTGCTCTACCTGCACCAGA

Table S1. Primers for CCL3/4 and IRF-1/6/7/8 mRNA detection. Six pairs of primers were designed to amplify rat CCL3/4 and IRF-1/6/7/8 mRNA. The sequence of these primers was shown.

Figure S1

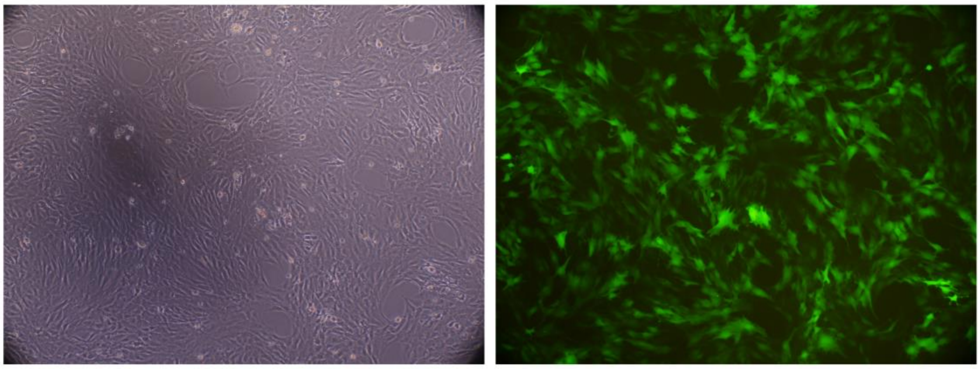

Figure S1. Transfection efficiency of shRNA expression plasmids into rat GMCs. Transient transfection of shCTR expression plasmids into the cultured GMCs was conducted with Neon^{TM} transfection system according to the manufacturer's procedure. The transfection efficiency of shRNA plasmids was detected by GFP at 48 h after transfection (right), and meanwhile, the same visual field were observed under ordinary light (left). The transfection efficiency was 80%~90% by counting the ratio of GFP expressing cells to total cells. The representative images were displayed (Magnification: ×100).

Figure S2

A

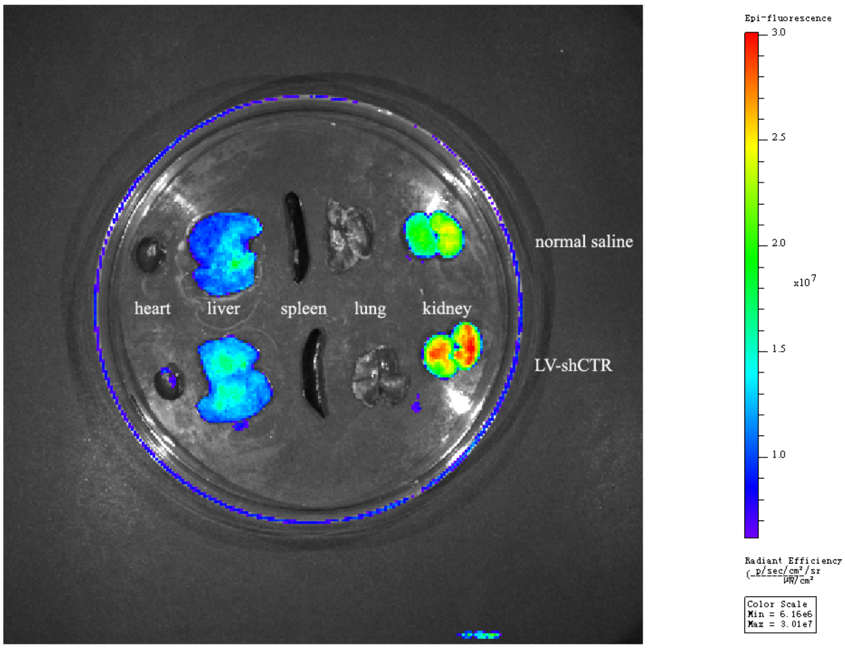

B

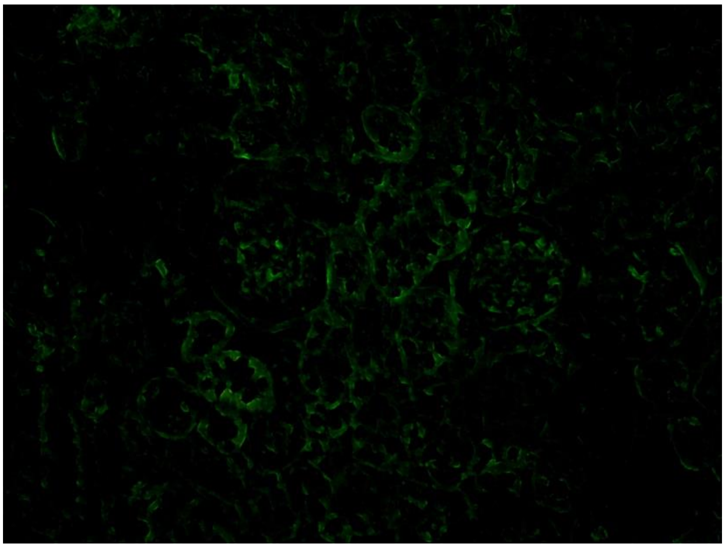

Figure S2. Transfection efficiency of LV-shRNA *in vivo*. The LV-shCTR was transfected into rat kidneys via renal artery perfusion suddenly followed by renal veins occlusion for 10 minutes. (A) At 96 h after perfusion, EGFP expression in different organs was examined by Caliper IVIS imaging system. (B) GFP expression in glomeruli and renal tubules was seen under a fluorescence microscope (Frozen sections, Magnification: ×100). The representative pictures were shown.

Figure S3

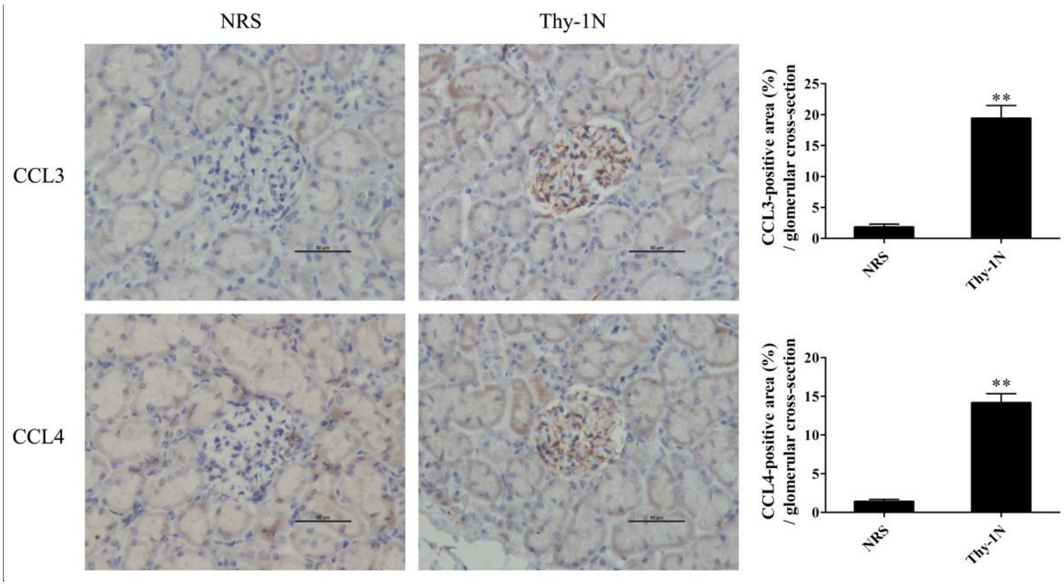

Figure S3. CCL3/4 expression in the renal tissues of rats. The protein levels of CCL3/4 in the renal tissues of Thy-1N and NRS rats at 6 h were examined by IHC staining (Magnification, ×400). ** P<0.01 vs. NRS group. Data were represented as means ± SE (n=5 in each group).

Figure S4

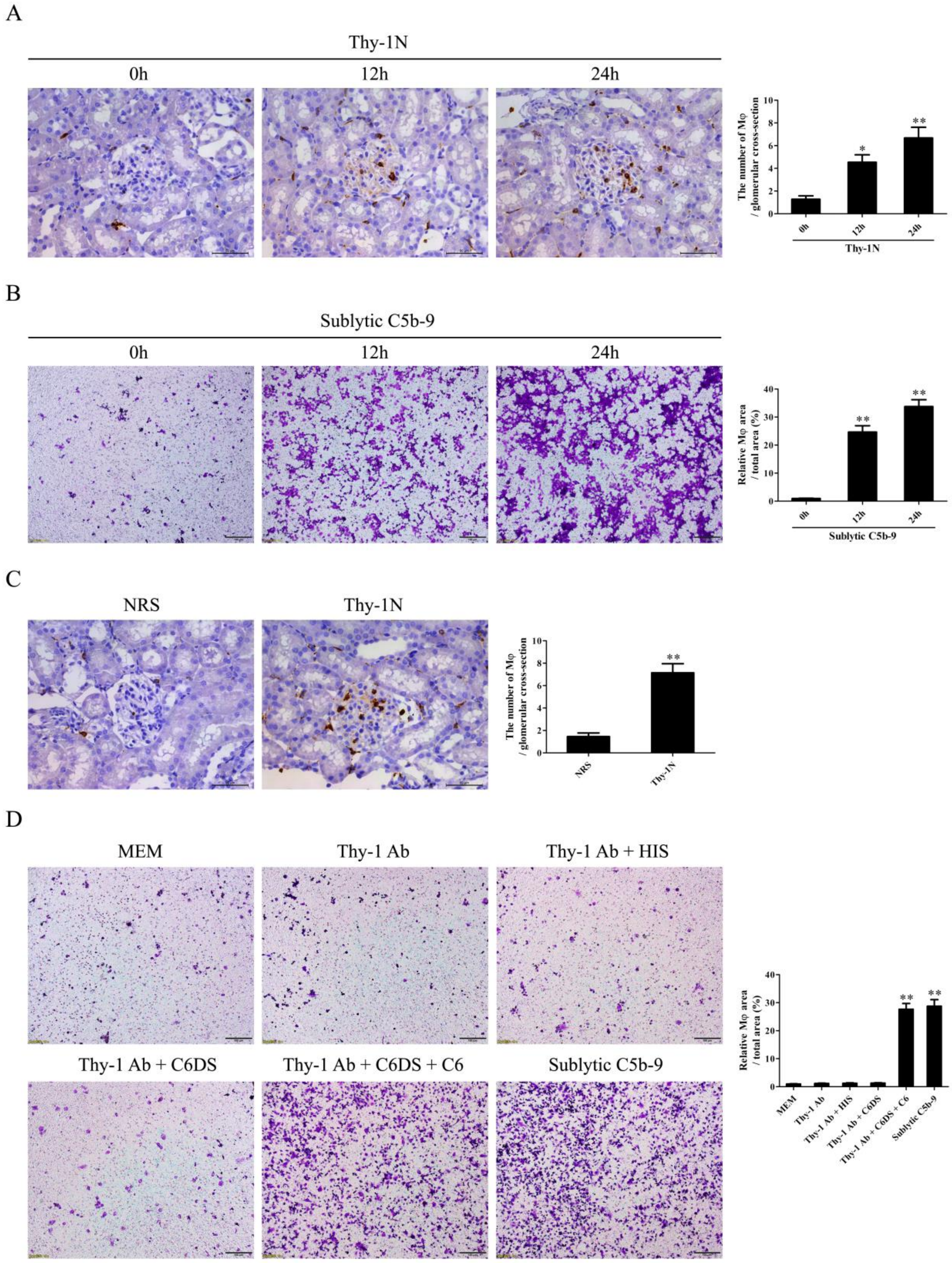

Figure S4. Mφ distribution in the renal tissues of Thy-1N rats and Mφ chemotaxis induced by GMCs exposed to sublytic C5b-9. (A) CD68^{+} cell (Mφ) in the renal tissues of Thy-1N rats at 0 h, 12 h and 24 h was examined by IHC staining with anti-CD68 antibody (Magnification: ×400). * P<0.05, ** P<0.01 vs. 0h. (B) An upper chamber containing Mφ

was separated by a membrane from a lower chamber containing GMCs following sublytic C5b-9 treatment for 0 h, 12 h and 24 h. Chemotaxis of Mφ from the upper chamber into the lower chamber was observed by crystal violet staining of the membrane (Magnification: ×100). ** $P<0.01$ vs. 0h. (C) Mφ in the renal tissues of Thy-1N rats and NRS rats at 24 h was observed by IHC staining with anti-CD68 antibody (Magnification: ×400). ** $P<0.01$ vs. NRS group. (D) An upper chamber containing Mφ was separated by a membrane from a lower chamber containing GMCs. GMCs were divided into different groups of MEM, Thy-1 Ab, Thy-1 Ab + HIS, Thy-1 Ab + C6DS, Thy-1 Ab + C6DS + C6 and sublytic C5b-9 (Thy-1 Ab + NHS) respectively. Chemotaxis of Mφ from the upper chamber into the lower chamber was observed by crystal violet staining of the membrane at 24 h after grouping treatment (Magnification: ×100). ** $P<0.01$ vs. other groups. Data were represented as means ± SE (n=5).

Figure S5

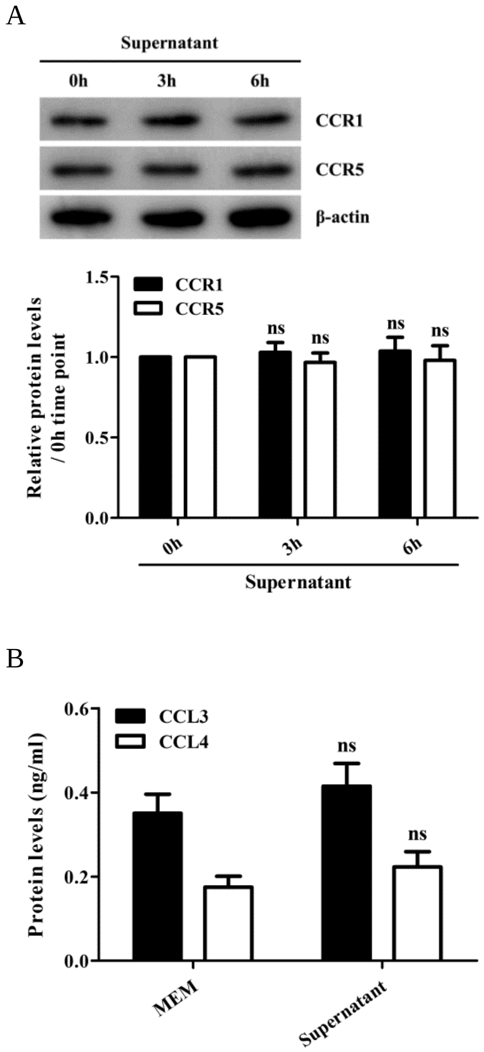

Figure S5. The expression or secretion of CCR1/5 and CCL3/4 in Mφ with the stimulation of supernatant from the GMCs induced by sublytic C5b-9. The GMCs were stimulated with sublytic C5b-9, and the supernatant was obtained after six hours. Mφ were incubated with the supernatant for 10 min, and changed to normal medium for continuous culture for 3 h and 6 h, and the expression of CCR1/5 and secretion of CCL3/4 were determined by IB (A, 3 h and 6 h) and ELISA (B, 6 h). Data were represented as means ± SE (n=3 in each group). ^{ns} P>0.05 vs. 0 h time point or MEM group.

Figure S6

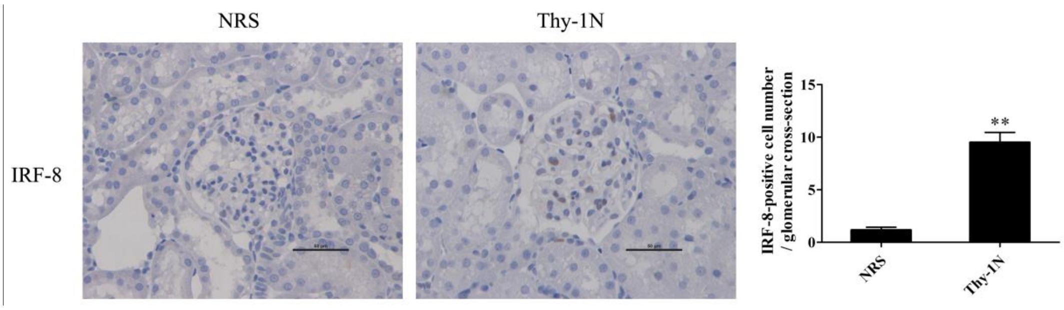

Figure S6. IRF-8 expression in the renal tissues of rats. The protein expression of IRF-8 in the renal tissues of Thy-1N and NRS rats at 3 h was examined by IHC staining (Magnification, ×400). ** P<0.01 vs. NRS group. Data were represented as means ± SE (n=5 in each group).

Figure S7

A

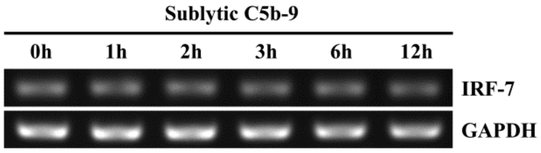

B

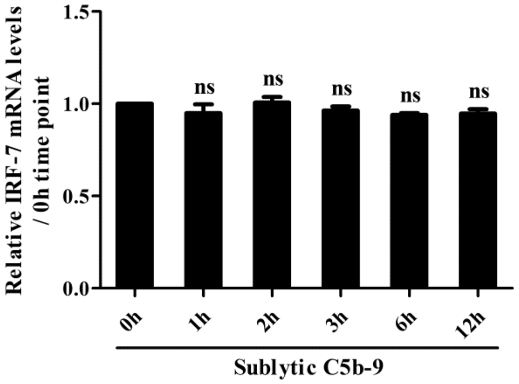

Figure S7. IRF-7 expression in the GMCs exposed to sublytic C5b-9. Rat GMCs were stimulated with sublytic C5b-9 for different time points, and then the mRNA levels of IRF-7 and GAPDH were detected by RT-PCR. Data were represented as means ± SE (n=3 in each group). [ns] P>0.05 vs. 0 h time point.

Figure S8

A

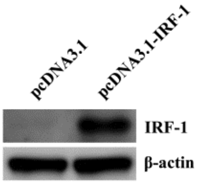

B

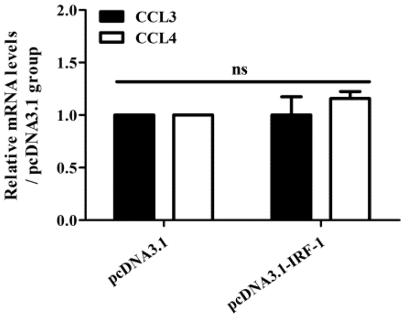

C

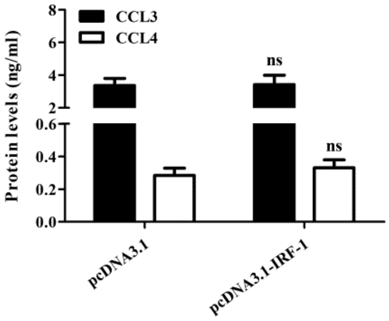

Figure S8. The effect of IRF-1 overexpression on CCL3/4 production in the GMCs. Rat GMCs were transfected with pcDNA3.1-IRF-1 or pcDNA3.1 for 48 h, and then the expression levels of IRF-1 and CCL3/4 in the GMCs were detected by IB (A), qPCR (B) and ELISA (C). Data were represented as means ± SE (n=3). ^{ns} P>0.05 vs. pcDNA3.1 group.

Figure S9

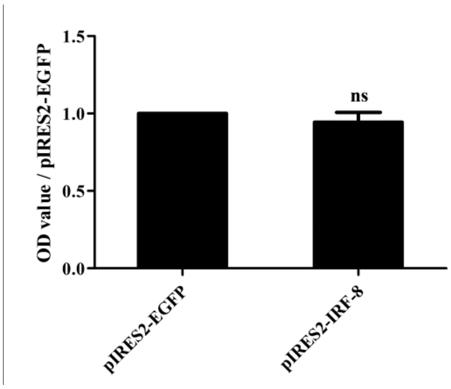

Figure S9. The effect of IRF-8 overexpression on GMC proliferation. Rat GMCs were transfected with pIRES2-IRF-8 and pIRES2-EGFP for 48 h, and the cellular proliferation was detected by CCK-8 assay. Data were represented as means ± SE (n=3). ^{ns} P>0.05 vs. pIRES2-EGFP group.

Figure S10

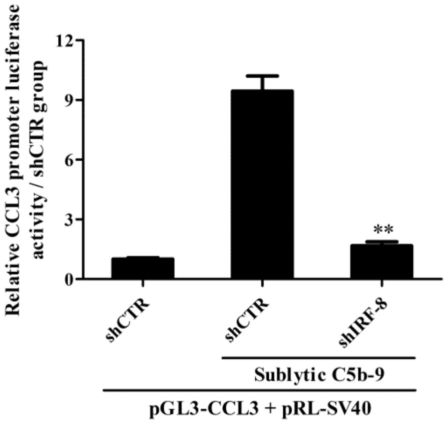

Figure S10. The effect of IRF-8 knockdown on CCL3 gene transcription in the GMCs induced by sublytic C5b-9. The plasmids of shIRF-8, shCTR, pGL3-CCL3 and pRL-SV40 were co-transfected into rat GMCs for 48 h in different groups followed by sublytic C5b-9 stimulation for 3 h, and then the luciferase activity was detected. Data were represented as means ± SE (n=3). ** P<0.01 vs. shCTR + sublytic C5b-9.

Figure S11

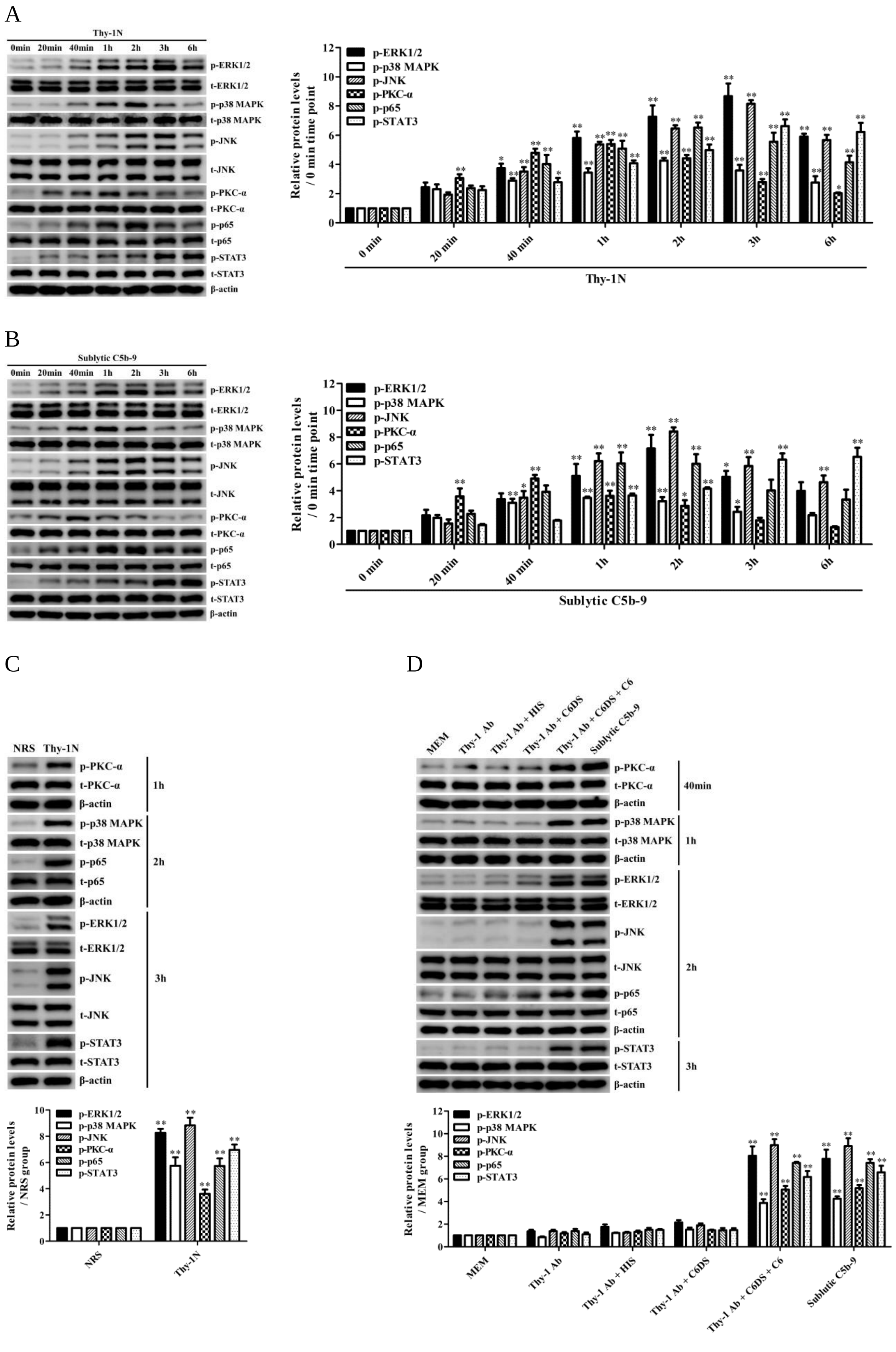
A
Thy-1N
0min 20min 40min 1h 2h 3h 6h
p-ERK1/2
t-ERK1/2
p-p38 MAPK
t-p38 MAPK
p-JNK
t-JNK
p-PKC-α
t-PKC-α
p-p65
t-p65
p-STAT3
t-STAT3
β-actin
Relative protein levels / 0 min time point
0 min 20 min 40 min 1h 2h 3h 6h
B
Sublytic C5b-9
C
NRS Thy-1N
1h
2h
3h
Relative protein levels / NRS group
D
MEM
Thy-1 Ab
Thy-1 Ab + HIS
Thy-1 Ab + C6DS
Thy-1 Ab + C6DS + C6
Sublytic C5b-9
40min
1h
2h
3h
Relative protein levels / MEM group

Figure S11. The expression and phosphorylation of ERK1/2, p38 MAPK, JNK, PKC-α, p65 and STAT3 *in vivo* and *in vitro*. (A and B) The expression and phosphorylation levels of ERK1/2, p38 MAPK, JNK, PKC-α, p65 and STAT3 in the renal tissues of Thy-1N rats (A) and in the GMCs exposed to sublytic C5b-9 (B) for different time was examined by IB. * $P<0.05$, ** $P<0.01$ vs. 0h. (C) The expression levels of above-mentioned proteins in the renal tissues of Thy-1N and NRS rats at 1 h, 2 h or 3 h were examined by IB. ** $P<0.01$ vs. NRS group. (D) Rat GMCs were divided into different groups. At 40 min, 1 h, 2 h or 3 h after treatment, the expression levels of above-mentioned proteins were determined by IB. ** $P<0.01$ vs. other groups. Data were represented as means ± SE (n=5 *in vivo*, n=3 *in vitro*).

Figure S12

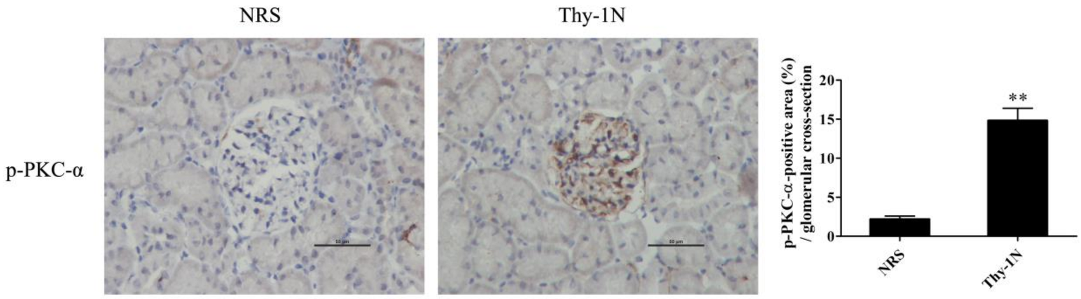

Figure S12. The phosphorylation of PKC-α in the renal tissues of rats. The protein level of p-PKC-α-Thr638 in the renal tissues of Thy-1N and NRS rats at 1 h was examined by IHC staining (Magnification, ×400). ** $P<0.01$ vs. NRS group. Data were represented as means ± SE (n=5 in each group).

Figure S13

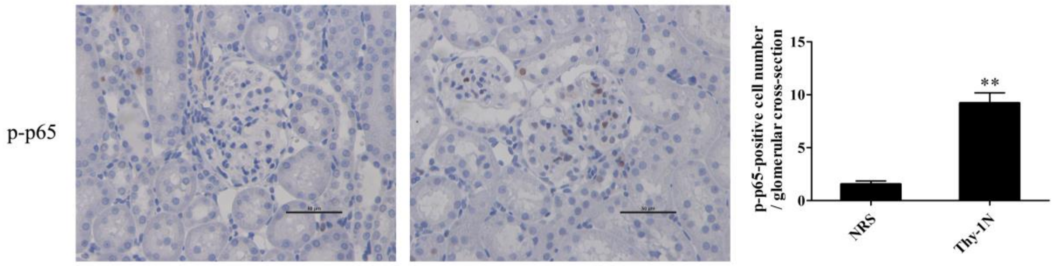

Figure S13. The phosphorylation of p65 in the renal tissues of rats. The protein levels of p-p65-Ser536 in the renal tissues of Thy-1N and NRS rats at 2 h were examined by IHC staining (Magnification, ×400). ** P<0.01 vs. NRS group. Data were represented as means ± SE (n=5 in each group).

Figure S14

A

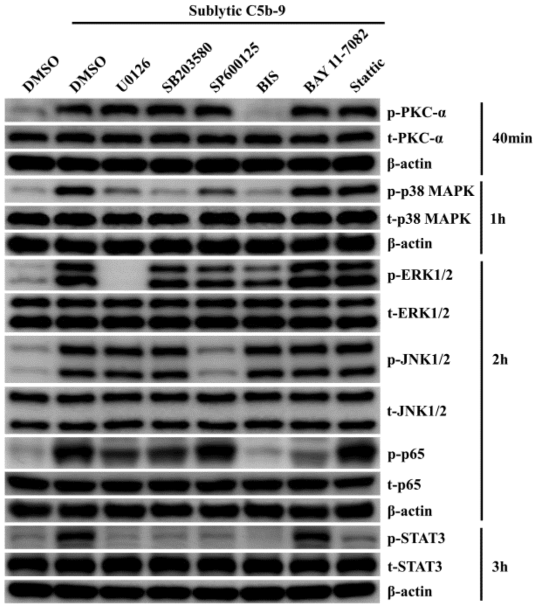

B

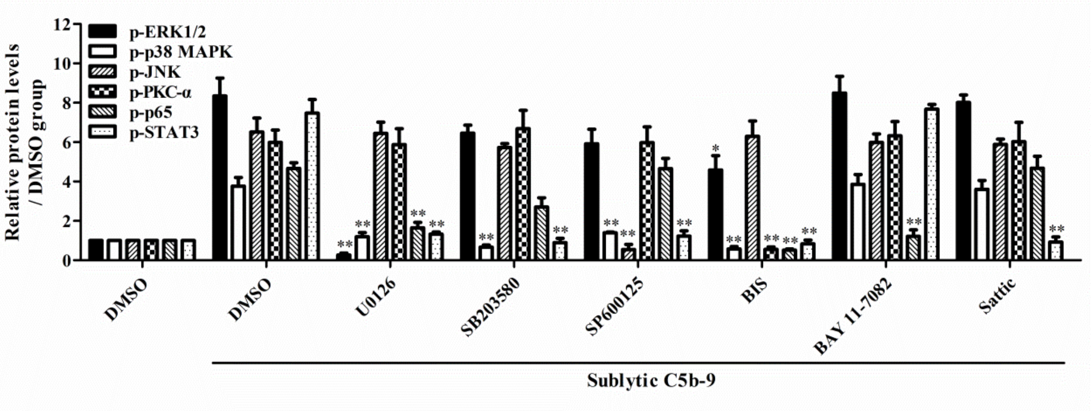

C

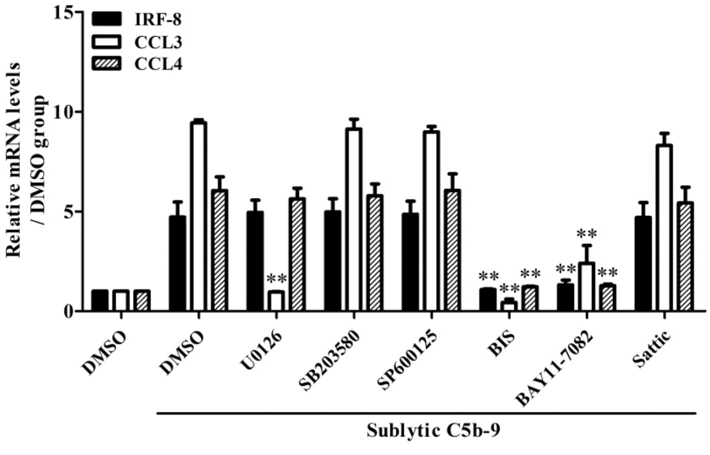

Figure S14. The effects of different inhibitors on the corresponding protein expression and CCL3/4 production. Rat GMCs was incubated with U0126 (ERK1/2 inhibitor, 10 μM), SB203580 (p38 MAPK inhibitor, 10 μM), SP600125 (JNK inhibitor, 10 μM), BIS (PKC-α inhibitor, 10 μM), BAY 11-7082 (p65 inhibitor, 10 μM), Stattic (STAT3 inhibitor, 10 μM) for 30 min, and then stimulated with sublytic C5b-9 for different time. The expression and phosphorylation levels of ERK1/2, p38 MAPK, JNK, PKC-α, p65, STAT3, IRF-8 and CCL3/4 were detected by IB (A and B) and qPCR (C). ** $P<0.01$ vs. DMSO + sublytic C5b-9 group. Data were represented as means ± SE (n=3).

Figure S15

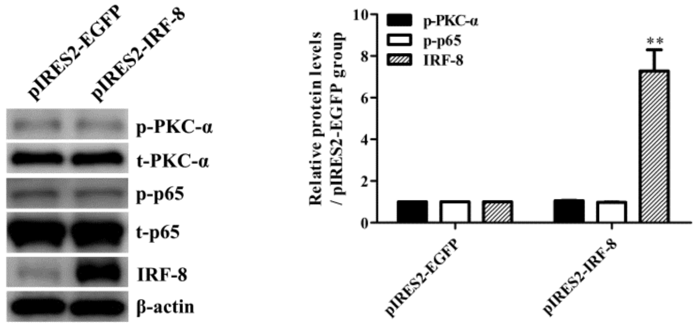

Figure S15. The effects of IRF-8 overexpression on PKC-α and p65 phosphorylation. Rat GMCs were transfected with pIRES2-IRF-8 or pIRES2-EGFP for 48 h, and then the expression and phosphorylation of IRF-8, PKC-α and p65 in the GMCs were detected by IB experiments. ** $P<0.01$ vs. pIRES2-EGFP group. Data were represented as means ± SE (n=3).

Figure S16

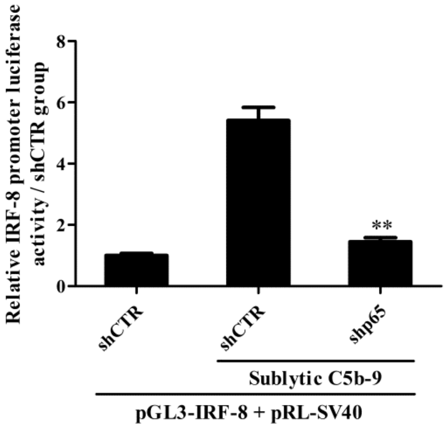

Figure S16. The effect of p65 knockdown on IRF-8 gene transcription in the GMCs induced by sublytic C5b-9. The plasmids of shp65, shCTR, pGL3-IRF-8 and pRL-SV40 were co-transfected into rat GMCs for 48 h in different groups followed by sublytic C5b-9 stimulation for 3 h, and then the luciferase activity was detected. Data were represented as means ± SE (n=3). ** P<0.01 vs. shCTR + sublytic C5b-9.

Figure S17

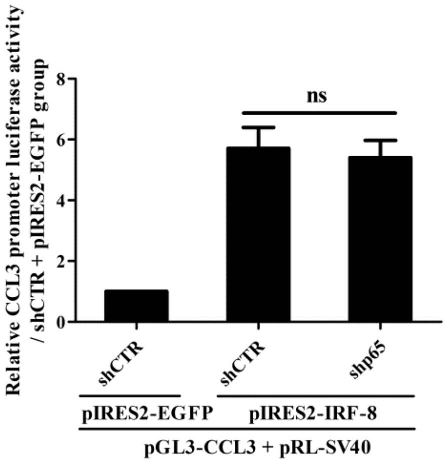

Figure S17. The effect of p65 knockdown on CCL3 gene transcription in the GMCs induced by IRF-8 overexpression. The plasmids of shp65, shCTR, pIRES2-IRF-8, pIRES2-EGFP, pGL3-CCL3 and pRL-SV40 were co-transfected into rat GMCs for 48 h in different groups, and then the luciferase activity was detected. Data were represented as means ± SE (n=3). ^{ns} P>0.05.

Figure S18

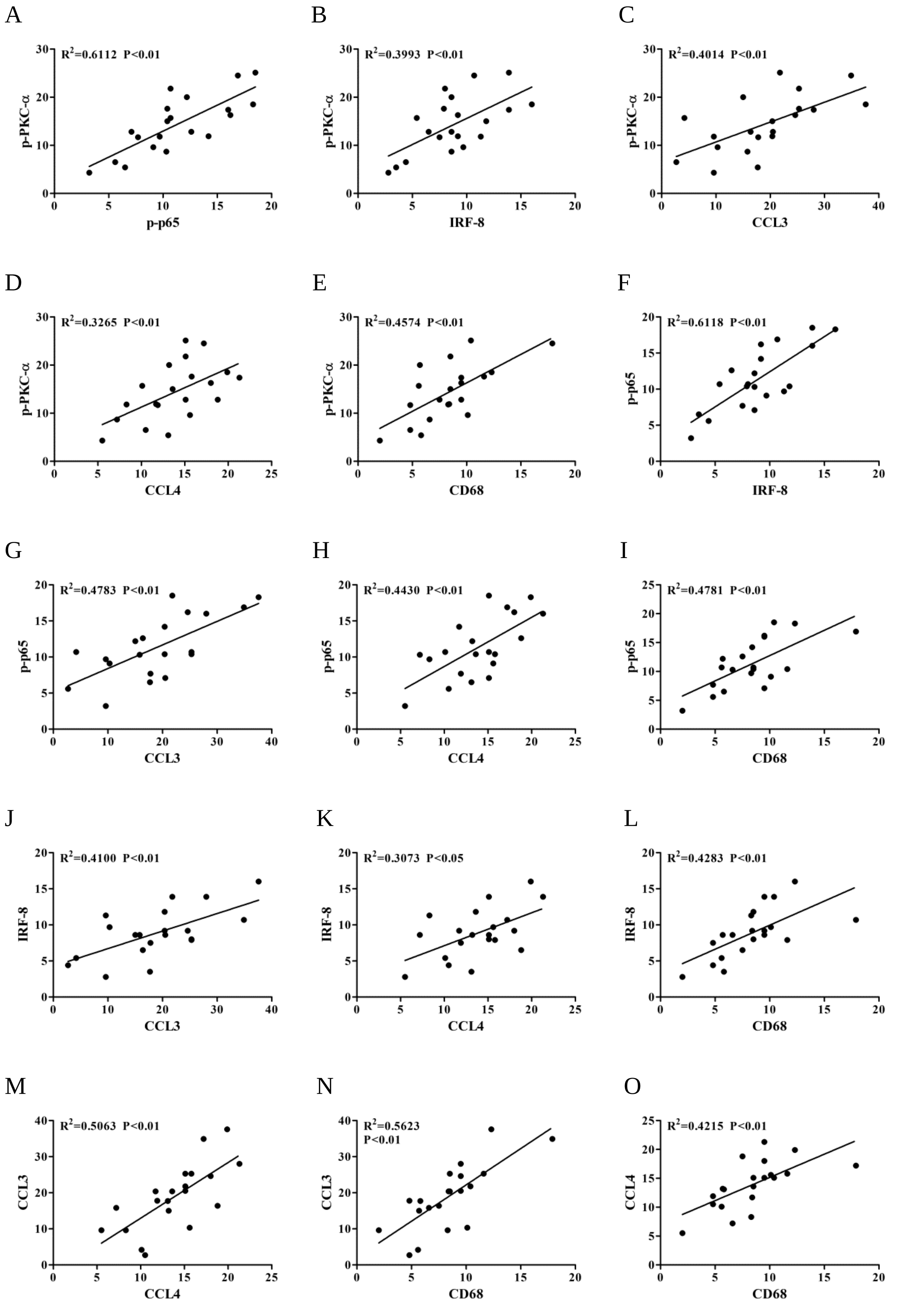

Figure S18. Correlation analysis between p-PCK-α, p-p65, IRF-8, CCL3/4 and CD68 protein expression in the renal tissues of MsPGN patients.
